# Pharmacological and natural products diversity of the brown algae genus *Sargassum*

**DOI:** 10.1039/d0ra03576a

**Published:** 2020-07-01

**Authors:** Mohammed I. Rushdi, Iman A. M. Abdel-Rahman, Hani Saber, Eman Zekry Attia, Wedad M. Abdelraheem, Hashem A. Madkour, Hossam M. Hassan, Abeer H. Elmaidomy, Usama Ramadan Abdelmohsen

**Affiliations:** Department of Pharmacognosy, Faculty of Pharmacy, South Valley University 83523 Qena Egypt; Department of Botany and Microbiology, Faculty of Science, South Valley University 83523 Qena Egypt; Department of Pharmacognosy, Faculty of Pharmacy, Minia University 61519 Minia Egypt usama.ramadan@mu.edu.eg +20-86-2369075 +20-86-2347759; Department of Medical Microbiology and Immunology, Faculty of Medicine, Minia University 61519 Minia Egypt; Department of Marine and Environmental Geology, National Institute of Oceanography and Fisheries Red Sea Branch 84511 Hurghada Egypt; Department of Pharmacognosy, Faculty of Pharmacy, Beni-Suef University Beni-Suef 62514 Egypt; Department of Pharmacognosy, Faculty of Pharmacy, Deraya University Universities Zone 61111 New Minia City Egypt

## Abstract

*Sargassum* (F. Sargassaceae) is an important seaweed excessively distributed in tropical and subtropical regions. Different species of *Sargassum* have folk applications in human nutrition and are considered a rich source of vitamins, carotenoids, proteins, and minerals. Many bioactive compounds chemically classified as terpenoids, sterols, sulfated polysaccharides, polyphenols, sargaquinoic acids, sargachromenol, and pheophytin were isolated from different *Sargassum* species. These isolated compounds and/or extracts exhibit diverse biological activities, including analgesic, anti-inflammatory, antioxidant, neuroprotective, anti-microbial, anti-tumor, fibrinolytic, immune-modulatory, anti-coagulant, hepatoprotective, and anti-viral activities. This review covers the literature from 1974 to 2020 on the genus *Sargassum*, and reveal the active components together with their biological activities according to their structure to create a base for additional studies on the clinical applications of *Sargassum*.

## Abstract 2

*Sargassum* (F. Sargassaceae) is an important seaweed excessively distributed in tropical and subtropical regions.

## Graphical abstract

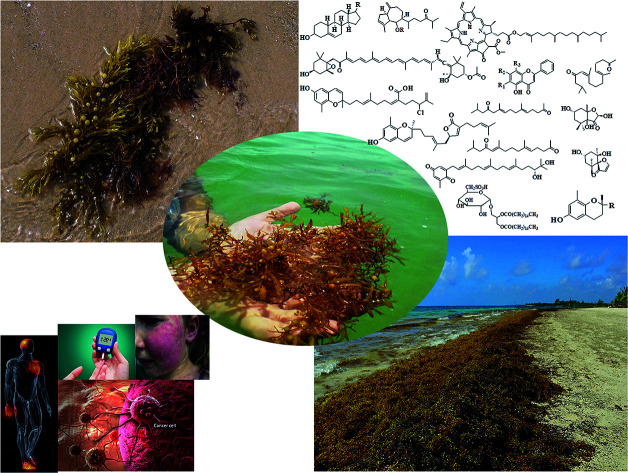

## 1. Introduction

Marine natural products have been characterized by their chemical structural diversity, along with different biological activities. The Red Sea is considered one of the most important marine hotspots comprising high biodiversity. Many marine algae are native to the Red Sea, which could be classified into brown algae (Phaeophyta), green algae (Chlorophyta), and red algae (Rhodophyta). Brown seaweeds are predominantly brown in color because of their contents of carotenoid fucoxanthin and polysaccharides, namely alginates, laminarins, fucans, and cellulose. Green seaweeds are characterized by their chlorophyll a and b content with ulvan being the major polysaccharide. While, in red seaweeds, the principal pigments are phycoerythrin, phycocyanin, and the primary polysaccharides are agars and carrageenans.^1^ Also, marine algae are rich sources of structurally diverse bioactive compounds with various biological activities, although only a few species have been chemically examined in the last decades.^1^

*Sargassum* (F. Sargassaceae), of the order Fucales, subclass Cyclosporeae, and class Phaeophyceae, is a genus of brown algae, communally known as gulfweed or sea holly, and is considered one of the most complex Phaeophyceae genera.^2^*Sargassum* was discovered by Agardh in 1820 and is reported to contain 537 species names in the algae database, of which 358 species have been accepted taxonomically.^2^ It comprises many different species that are distributed worldwide, although primarily found in tropical and subtropical marine waters, and also generally growing on rocky reefs.

**Fig. 1:**
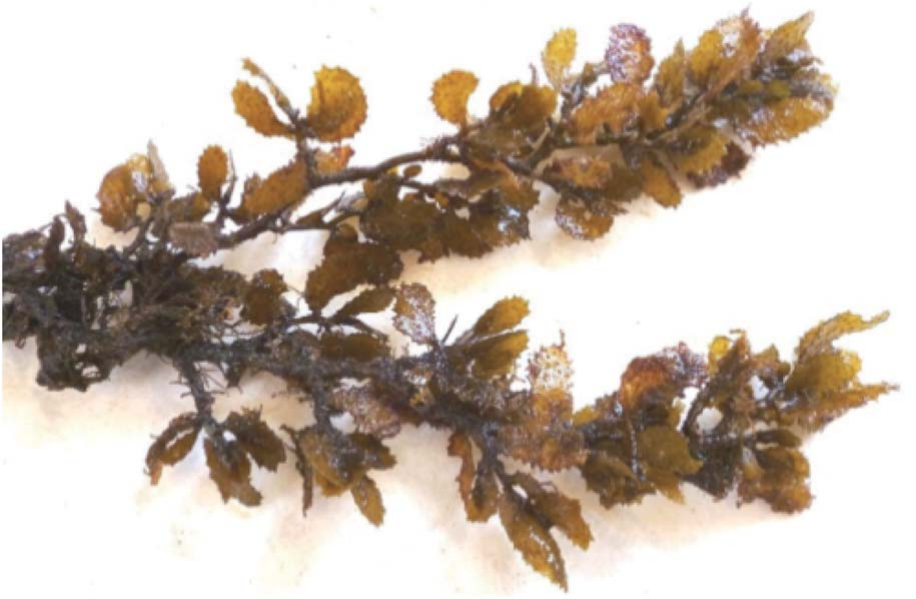
*Sargassum sanyaense* from Hurghada City coast line, Red Sea (Egypt).

*Sargassum* thallus (10–200 cm) may be linear or bushy, with stipes 1–20 cm long arising from a discoid–conical holdfast, and do not penetrate the substratum. Its main axes are perennial, short, and cylindrical or flattened in sections, and bear the scars of deciduous branches. The shape of the leaves can be simple, bifid, or divided several times, round, spatulate, turbinate, lanceolate, ovoid, linear or of any intermediate form with air bladders (vesicles) normally present, subspherical to the ovoid, petiolate, mutic or apiculate, replacing the ramuli or axillary to the laterals. The basis of the leaves is rounded or attenuate, symmetrical or not. The pedicel may be variable in length, cylindrical, or flattened in sections and smooth. The leaves margin may be simple or doubled at the apex, and may be smooth, undulated, finely serrated, deeply dentated, or any intermediate aspect. The midrib may be short and thick or thinner and reaching the apex or any intermediate length. The apex may be acute, rounded, or truncated, simple, or showing a cup-like shaped depression (Fig. 1).^2^

A survey of folk uses of *Sargassum* spp. in the last 20 years period (1977–1996), showed that different regions in the tropical country use the brown seaweed differently. Commonly, *Sargassum* spp., is used as a cover or wrapper of fish to maintain their freshness. In the Ilocos Region in the Northern Philippines, *Sargassum* spp. is used as a vegetable; whereas in the Visayas and Northern Mindanao, natives utilize *Sargassum* spp. as a fertilizer, flower inducer, and insect repellant. The Boholano people also use the brown seaweed as animal feed. In certain parts of the island of Bohol, a *Sargassum* drink is made and is reported to have health benefits.^1^

For nearly 2000 years, *Sargassum* spp. has been also used in Traditional Chinese Medicine (TCM) to treat a variety of diseases, including thyroid.^1^ Although *Sargassum* has not attracted the attention of most researchers, the therapeutic potentials of pure compounds isolated from *Sargassum* are promising for their antiviral, antimicrobial, cytotoxic, antipyretic, anti-inflammatory, cardioprotective, hepatoprotective, and hypolipidemic properties.^3^ Some metabolites, such as embelin, tanacetol A, and oxygenated fucosterols, have attracted great attention because of their uncommon structural complexity and interesting pharmacological properties.^1,3^ Some species are economically important, especially in the food, textile, cosmetics, and pharmaceutical industries. This review covers the literature on the genus *Sargassum* along with its chemical and medicinal potential.

## 2. Sargassum angustifolium

*S*. *angustifolium*, commonly known as narrow leaf *Sargassum* weed, is widely distributed around the Western Indian Ocean.^4^ The *n*-hexane, dichloromethane, and *n*-butanol fractions isolated from *S. angustifolium*, collected from the Persian Gulf, were reported to have a cytotoxic effect against HeLa (cervical cancer) and MCF-7 (breast cancer) cells, where the cell survival was inversely proportional to the increase in the concentration of the different extracts, respectively, from 150 μg mL^−1^ to 900 μg mL^−1^. The, *n*-hexane fraction was shown to have a median growth inhibitory concentration value (MIC) of 71 and 77 μg mL^−1^, against HeLa and MCF-7, while the MIC of the dichloromethane fraction was 36 and 88 μg mL^−1^, respectively. The *n*-butanol fraction showed an MIC of 25 μg mL^−1^ against MCF-7.^4^ Sulfated polysaccharides isolated from *S. angustifolium* were reported to have potent immuno-stimulant activity, through inducing RAW264.7 macrophage cells to release nitric oxide, IL-1β, TNF-α, IL-6, IL-10, and IL-12 through activation of the NF-κB and MAPKs signaling pathways.^5^ Also, the phosphate buffer extract of *S. angustifolium* had a low α-amylase inhibition activity with an IC_50_ of 1.85 mg mL^−1^.^6^

## 3. Sargassum aquifolium

**Fig. 2:**
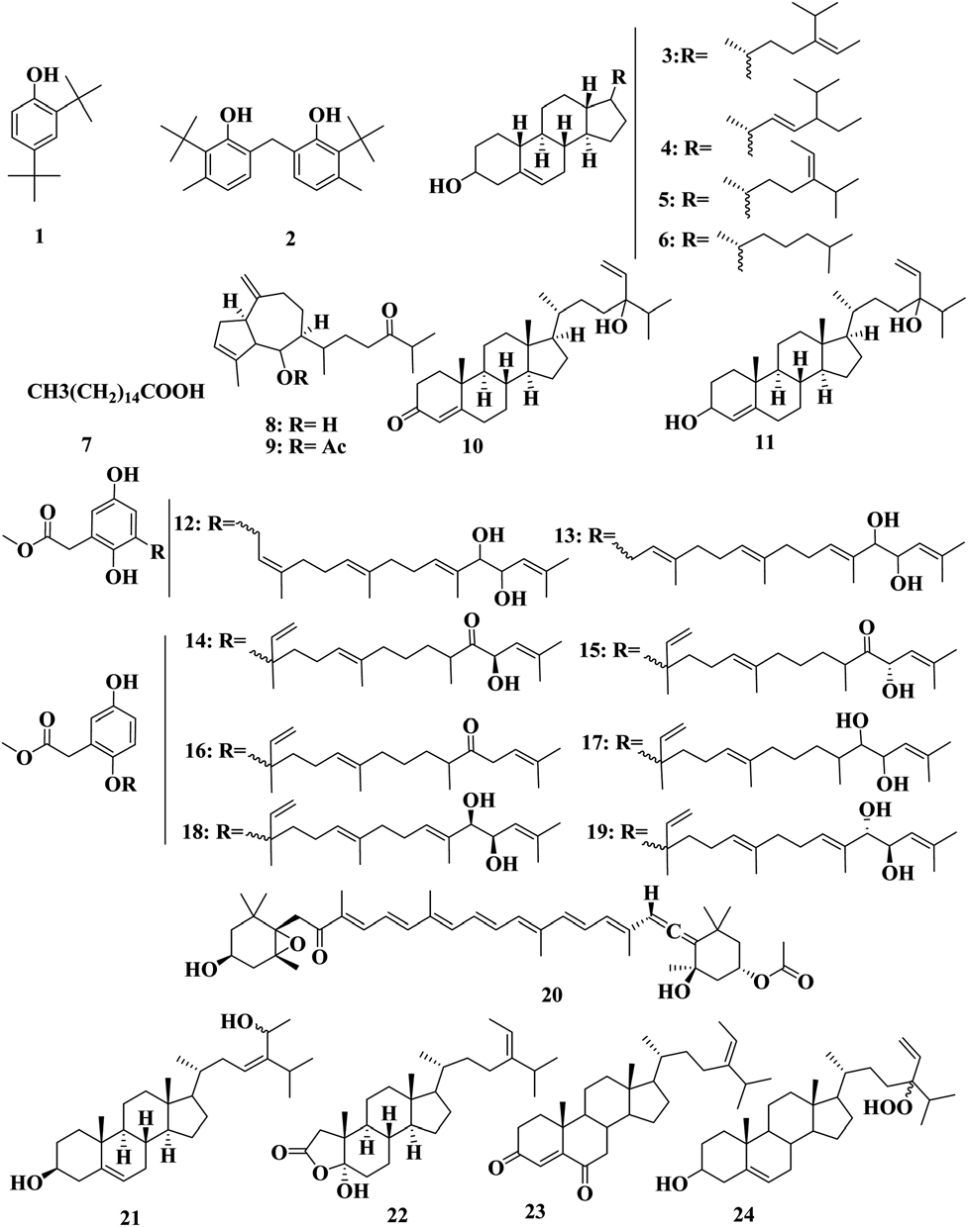
Chemical structures of compounds 1–24.

GC-MS of a crude petroleum ether extract of *S. aquifolium* (Turner), collected from the Red Sea of Jazan (KSA), was reported containing phenol, 2,4-bis(1,1-dimethylethyl) 1, phenol, 2,2′-methylenebis[6-(1,1-dimethylethyl)-4-methyl 2, stigmasta-5,24(28)-dien-3-ol, (3*á*,24*Z*) 3, and stigmasterol 4 (Fig. 2).^7^ The crystals of a petroleum ether extract of *S. aquifolium* dissolved in Millipore water were tested for antibacterial activity against *S. aureus*, *S. pyogenes*, *B. subtilis*, *E. coli*, *P*. *aeruginosa*, and *K. pneumonia*. The maximum antibacterial activity was exerted against *E. coli*, with an MIC of 100–150 mg mL^−1^.^7^l-Fucose, d-galactose, d-mannose, d-glucuronic acid, d-xylose, and sulfate were found to be the main constituents of fucoidan detected in *S*. *aquifolium*, collected from Vietnam, with anticoagulant, cytotoxic, and antitumor activities, which increase with sulfation.^8^

## 4. Sargassum asperifolium

The polysaccharide extracts of *S. asperifolium*, collected from the Red Sea (Egypt), possessed anti-inflammatory activity, through the significant inhibition of nitric oxide generation, and LPS-induced TNF-α.^9^ Analysis of the volatile fraction of *S. asperifolium* led to the identification of fucosterol 5, cholesterol 6, and palmitic acid 7.^10^ Dictyone 8, dictyone acetate 9, saringosterone 10, and saringosterol 11 were also detected in *S. asperifolium* collected from Hurghada (Egypt) (Fig. 2).^11^

## 5. Sargassum autumnale

Isonahocol D1 12, D2 13, nahocol-A 14, nahocol-A1 15, nahocol-B, C, D1, and D2 16–19, were detected in a methanol extract of *S. autumnale*, collected from Omosu Bay (Japan). Nahocols were shown to have an aryl prenyl ether structure, which is considered a precursor of the prenyl hydroquinones or prenyl benzoquinones in the plant and animal kingdoms.^12^

## 6. Sargassum binderi

Fucoxanthin 20 was detected in Malaysian *S. binderi* and *S. duplicatum*. The amount of fucoxanthin 20 and the total lipid contents of *S. duplicatum* (1.01 ± 0.10 and 21.3 ± 0.10 mg g^−1^ dry weight, respectively) were significantly higher than those of *S. binderi* (0.73 ± 0.39 and 16.6 ± 4.10, respectively). Docosahexanoic acid, eicosapentanoic acid, arachidonic acid, linoleic acid and α-linolenic unsaturated fatty contents in *S. duplicatum* were found to be higher (0.76%, 2.55%, 13.64%, 5.81%, and 5.35%, respectively) than in *S. binderi* (0.70%, 1.82%, 9.13%, 6.37% and 4.39%, respectively), while palmitic acid (7) the major fatty acid in both samples.^13^

## 7. Sargassum boveanum

The different fractions of *n*-hexane, trichloroethane, chloroform, and *n*-butanol of the methanol-ethyl acetate extract of *S. boveanum*, collected from the Persian Gulf, showed a decrease in the cell survival of HeLa cells with increasing the concentration of the extracts, with IC_50_ values of 150.3 ± 23.10, 437.0 ± 147.3, 110.4 ± 33.67, and 1025.0 ± 15.20 μg mL^−1^, respectively.^14^

## 8. Sargassum carpophyllum

**Fig. 3:**
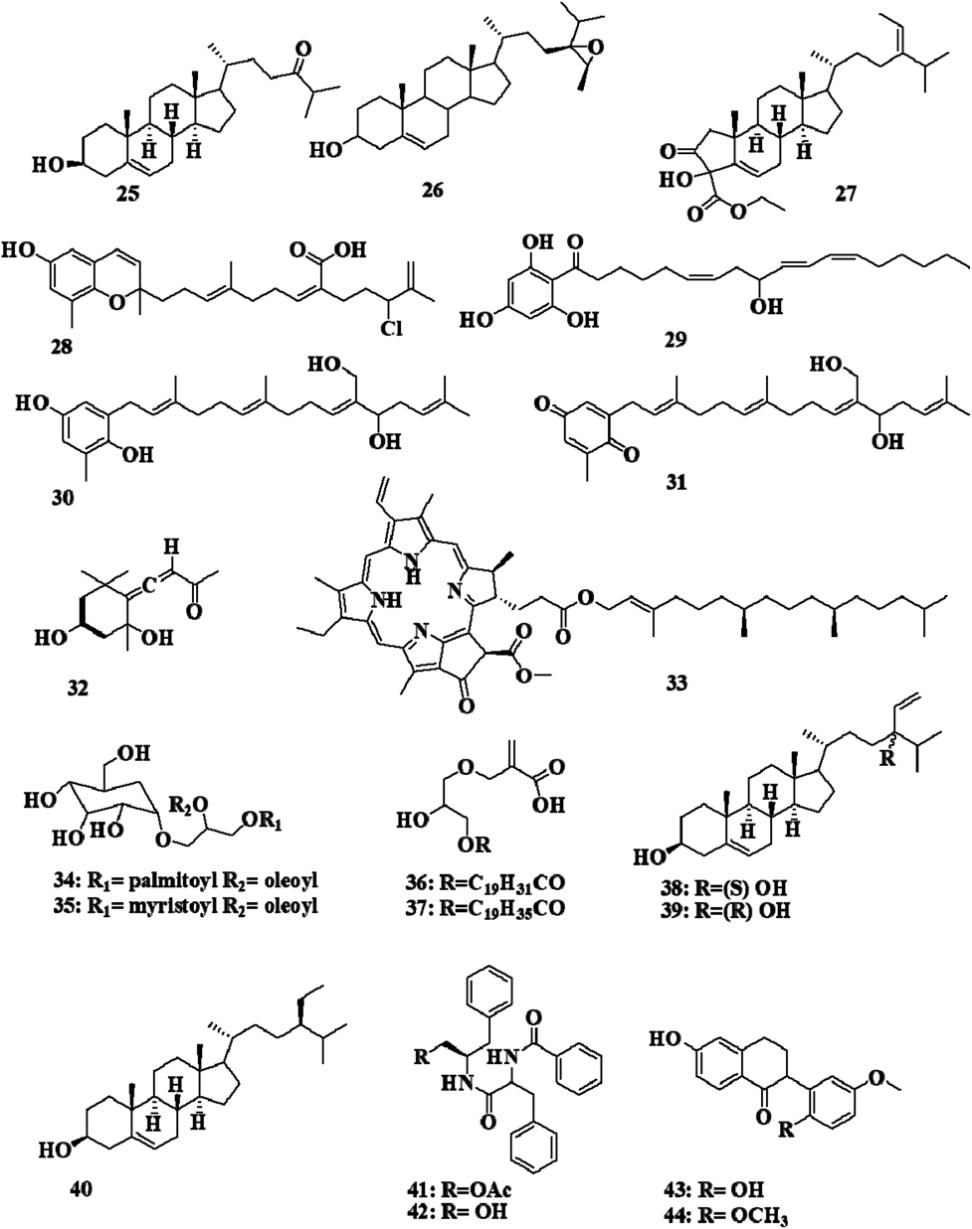
Chemical structures of compounds 21–44.

28ξ-Dihydroxy-24-ethylcholesta-5,23-*Z*-dien 21 and 2*a*-oxa-2-oxo-5*f*-hydroxy-3,4-dinor-24-ethylcholesta-24(28)-ene 22 together with five steroids, namely fucosterol 5, 24-ethylcholesta-4,24(28)-dien-3,6-dione 23, 24ξ-hydroperoxy-24-vinylcholesterol 24, 24-ketocholesterol 25, and 24*R*,28*R*- and 24*S*,28*S*-epoxy-24-ethylcholesterol 26 (Fig. 2 and 3) were detected in an ethanol extract of *S. carpophyllum*, collected from the Beihai (China) Sea. Compounds 21 and 24 reportedly had cytotoxic activities against HL-60, with IC_50_ values of 7.8 and 8.5 μg mL^−1^, respectively, while compounds 5 and 23 showed potent cytotoxic activities against P-388, with IC_50_ values of 0.7 and 0.8 μg mL^−1^, respectively. Compound 26 showed cytotoxic activity against MCF-7, HCT-8, 1A9, HOS, and PC-3, with IC_50_ values of 4.0, 8.8, 10.0, 10.0, and 7.2 μg mL^−1^, respectively.^15^ Another compound, 24-ethyl-3-carbethoxy-3-hydroxy-A-nor-cholesta-5,24(28)-dien-2-one 27, was also detected in *S. carpophyllum*, collected from the South China Sea, which was the first reported non-steroidal ester from natural sources.^16^

## 9. Sargassum cinereum

Fucoidan was detected in *S. cinereum*, collected from Tuticorin Lat. (India). Fucoidan was reported to contain 65.753% of l-fucose and 3.7% ± 1.54% of sulfate, respectively, with a monosaccharide composition, such as l-fucose, d-galactose, d-mannose, and d-xylose. The maximum 2,2′-diphenyl-1-picrylhydrazyl (DPPH) scavenger activity for the fucoidan extract was found at the concentration of 100 μg, whereas the crude extract of *S. cinereum* showed 63.58% ± 0.56% scavenging activity. Fucoidan extract showed 50% cell death after 24 h of incubation (75 ± 0.9037 μg mL^−1^) against the colon cancer cell line HCT-15.^17^ The mechanism of fucoidan was a dose-dependent inhibited growth of the colon cancer cell line Caco-2, (IC_50_ = 250 μg mL^−1^), the increased production of reactive oxygen species (ROS), and augmented mitochondrial membrane permeability.^18^

## 10. Sargassum crassifolium

An *S. crassifolium* ethanol extract, collected from Diora-Zinungan, showed antimicrobial activity against *A. hydrophila*, with an MIC value of 24.33 ± 0.58 mm.^19^

## 11. Sargassum cristaefolium

Sodium alginate was detected in *S. cristaefolium*, collected from Poteran Island (Indonesia). This alginate had a molecular weight of 217.94 ± 7.14 kDa with a mannuronic acid to guluronic acid ratio (M/G) of 0.28, and the l-guluronic acid block was 0.78, which was higher than the d-mannuronic acid block.^20^

## 12. Sargassum dentifolium

Polysaccharide extracts of *S. dentifolium* collected from the Red Sea (Egypt) had a protective effect against cyclophosphamide (CP)-induced genotoxicity in mice bone marrow cells (BMCs).^21^ Also, the ethanol extract of *S. dentifolium*, reported as hepatoprotective in carbon tetrachloride (CCl_4_)-induced hepatitis in rats, had antioxidant activity.^22^

## 13. Sargassum fallax

Fallachromenoic acid 28, an unusual halogenated meroditerpenoid, retroflexanone 29, fallahydroquinone 30, and fallaquinone 31 (Fig. 3), were isolated from the Southern Australian *S. fallax*, and were shown to have antitumor activities against a P388 Murine Leukaemia cell line, with an IC_50_ > 27–29 μM.^23^

## 14. Sargassum filipendula

Heterofucan extracted from *S. filipendula* was shown to have antiproliferative and apoptosis activities against human cervical cancer (HeLa) cells, at a concentration of 0.1 to 2.0 mg mL^−1^.^24^

## 15. Sargassum fluitans

The *in vivo* anti-fibrotic effect of a fucoidan extract of *S. fluitans* (chemically composed of carbohydrates, sulfates, uronic acids, protein, and phenols), collected from Puerto Morelos (Mexico), was detected in a CCl_4_-induced liver damage model in rats. The daily oral administration of fucoidan extract (50 mg kg^−1^) showed a significant reduction in liver enzymatic activity, liver infiltration of inflammatory cells, collagen fiber deposition, and gene expression cytokines.^25^

## 16. Sargassum fulvellum

Grasshopper ketone 32 was detected in an ethanol extract of *S. fulvellum* (SFEE) collected from Jindo (Korea). Grasshopper ketone 32 and SFEE were investigated on 2,4-dinitrochlorobenzene (DNCB)-induced atopic dermatitis (AD)-like skin lesions in BALB/c mice. SFEE and grasshopper ketone 32 were found to have an inhibitory effect on AD by regulating immune mediators and cells and thus may be a potentially effective alternative therapy for AD.^26^ Boiling water, ethanol, and dichloromethane extracts of *S. fulvellum*, collected from Wando aquaculture farm (Korea), were tested for anti-inflammatory, antipyretic, and analgesic activities in mice. The dichloromethane extract (0.4 mg per ear) significantly inhibited the inflammatory symptoms of mouse ear edema by 79.1%.^27^ Pheophytin A 33 was detected in *S. fulvellum* collected from the Japanese coastline. When PC12 cells were treated with a low concentration of pheophytin A 33 (3.9 μg mL^−1^) in the presence of a low level of nerve growth factor (10 ng mL^−1^), the compound produced neuritis outgrowth similar to that produced by a high level of nerve growth factor (50 ng mL^−1^). Pheophytin A 33 also enhanced signal transduction in the mitogen-activated protein kinase signaling pathway, which is also induced by nerve growth factor.^28^ 1-*O*-Palmitoyl-2-*O*-oleoyl-3-*O*-(α-d-glucopyranosyl)glycerol (POGG) 34 and 1-*O*-myristoyl-2-*O*-oleoyl-3-*O*-(α-d-glucopyranosyl)-glycerol (MOGG) 35 were obtained from *S. fulvellum* collected from the East Sea in China. POGG 34 and MOGG 35 showed fibrinolytic activity in the reaction system of pro-u-PA and plasminogen.^29^ Fulvellic acid esters 36 and 37 are glycerides bearing a methacrylic acid moiety and were detected in a methanol extract of *S. fulvellum* collected from Awakominato (Chiba) (Fig. 3).^30^

## 17. Sargassum fusiforme

The polysaccharide fraction detected in *S. fusiforme* collected from Qingdao (China) consisted of l-fucose, d-mannose, l-rhamnose, d-glucose, d-galactose, and d-glucuronic acid with different molar ratios. Regarding the hypoglycemic and hypolipidemic effects, the oral administration of these polysaccharide fractions prominently restrained the loss of body weight and increase of water intake, and also significantly controlled the increase in the levels of fasting blood glucose of diabetic rats, as well as showed better effects in controlling fasting blood glucose, alanine aminotransferase (ALT), uric acid (UA) and urea nitrogen (BUN) levels.^31^ The polysaccharide also showed a significant protective effect against ultraviolet B (UVB) radiation in hairless Kun Ming (KM) mice.^32^

## 18. Sargassum glaucescens

Stigmasterol 4, fucosterol 5, cholesterol 6, 24(*S*)-hydroxy-24-vinylcholesterol 38, 24(*R*)-hydroxy-24-vinylcholesterol 39, and β-sitosterol 40 (Fig. 3) were detected in *S. glaucescens* collected from Southern Iran. *In vitro* α-amylase inhibitory activities were detected with a methanol extract, whereby a potent inhibition (IC_50_ = 8.9 ± 2.4 mg mL^−1^) of the enzyme compared to acarbose as a positive control was detected.^33^ Fucoidan was detected in *S. glaucescens* collected from Kenting (Taiwan), where the monosaccharide composition was l-fucose, sulfate, and uronic acid, and it was also shown to exhibit antioxidant activities, in a dose-dependent manner, against DPPH.^34^

## 19. Sargassum hemiphyllum

A hot-water extract of *S. hemiphyllum* collected from the coast of Penghu County (Taiwan) was found to have antioxidant activity and immune-stimulating activities when using HB4C5, and J774.1 cells. The antioxidant activity increased with a concentration <3.5 mg mL^−1^. The HB4C5 cells showed the maximum relative activities of cell proliferation (174%) and IgM secretion (132%) with 120 μg mL^−1^ of the hot-water extract at 80 μg mL^−1^, while J774.1 cells showed the maximum relative activities of cell proliferation (141%) and phagocytosis (147%).^35^ The major polyphenols (17.35 ± 0.93–36.66 ± 2.01 mg g^−1^) were extracted using water, ethanol, and acetone (WES, EES, and AES, respectively) for *S. hemiphyllum*. The inhibition of α-amylase, α-glucosidase, sucrose, and maltase activities and stimulation of insulin secretion were greater with AES than with WES or EES. Moreover, 250 μg mL^−1^ EES and AES significantly increased insulin secretion in the presence of 25 mg mL^−1^ glibenclamide compared to with 50 mg mL^−1^ glibenclamide.^36^ Sulfated polysaccharide extracted from *S. hemiphyllum* (SHSP) was detected on the mouse macrophage cell line (RAW 264.7) activated by lipopolysaccharide (LPS), which can used as a model system. The secretion profiles of pro-inflammatory cytokines, including IL-1β, IL-6, TNF-α, and NO, were found to be significantly reduced in 1–5 mg mL^−1^ dose ranges of SHSP treatments, in which the anti-inflammatory properties of SHSP may be attributed to the downregulation of NF-κB in the nucleus.^37^

## 20. Sargassum henslowianum

**Fig. 4:**
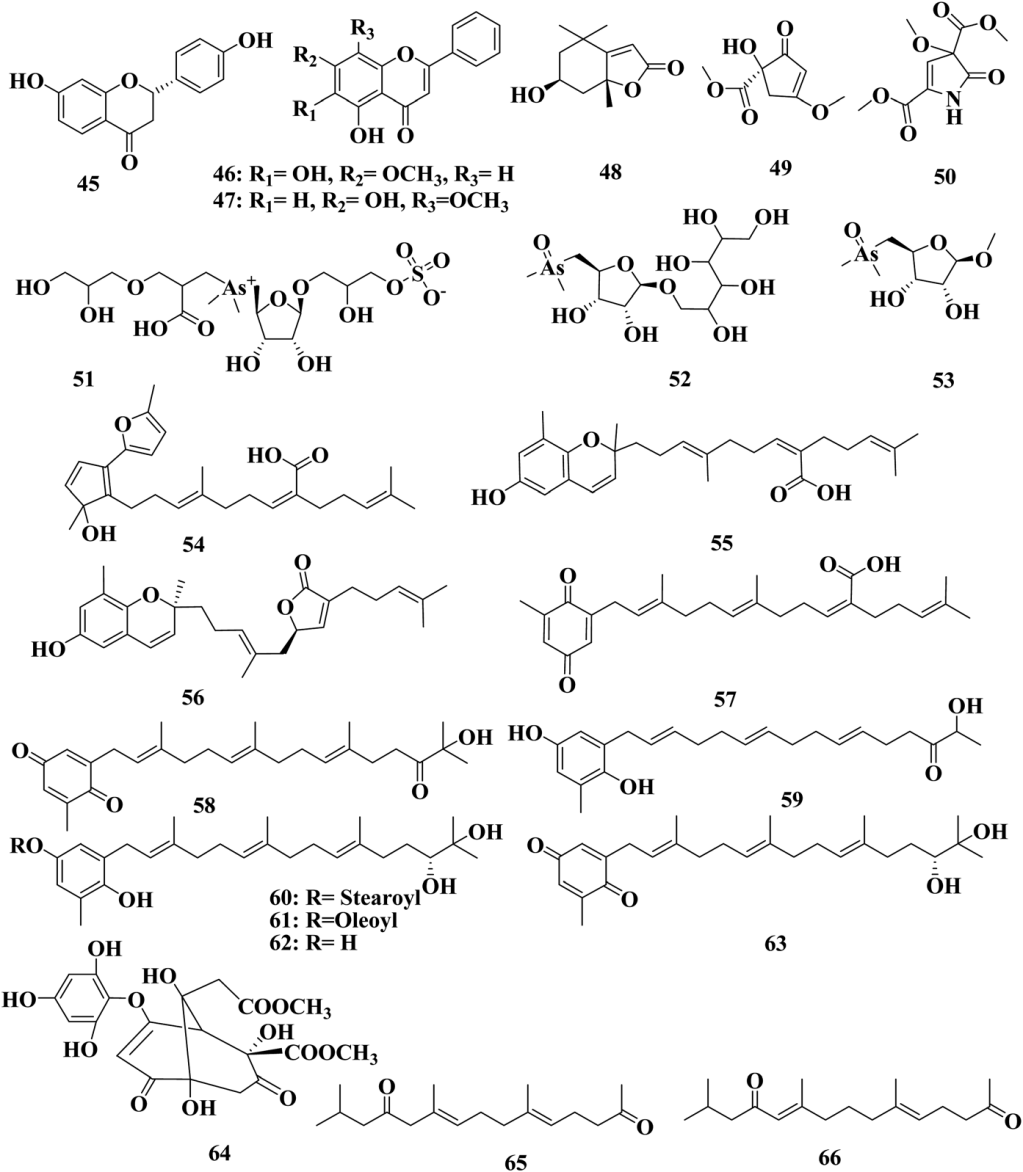
Chemical structures of compounds 45–66.

Saringosterol 11, aurantiamide acetate 41, aurantiamide 42, vestitone 43, 7-hydroxy-2′,4′-dimethoxyisoflavanone 44, liquiritigenin 45, 5,6-dihydroxy-7-methoxyflavone 46, and 5,7-dihydroxy-8-methoxyflavone 47 (Fig. 3 and 4) were detected in an ethanol extract of *S. henslowianum* collected from Guangdong and Fujian Provinces (China).^38^ Fucoidan was also detected in *S. henslowianum* collected from Hai Van–Son Cha peninsula (Vietnam). The oral dose (100 mg kg^−1^) of fucoidan was shown to decrease cholesterol, triglyceride, and LDL-cholesterol levels in obese mice.^39^ Fucose-containing sulfated polysaccharides (FCSPs) extracted from *S. henslowianum* notably had dose-dependent growth-inhibitory effects on the proliferation of melanoma B16 cells.^40^

## 21. Sargassum horneri

The anti-inflammatory effects of an ethanol extract of *S. horneri* (ESH) on the RAW 264.7 murine macrophage cell line were tested. ESH was not cytotoxic to RAW 264.7, while a dose of ESH 200 μg mL^−1^ was shown to reduce the mRNA level of cytokines, including IL-1β, and pro-inflammatory genes as iNOS and COX-2 in LPS-stimulated macrophage activation. ESH was also found to elicit anti-inflammatory effects by inhibiting ERK, p-p38, and NF-κB phosphorylation.^41^ Also, 6-hydroxy-4,4,7*a*-trimethyl-5,6,7,7*a*-tetrahydrobenzofuran-2(4*H*)-one 48 (Fig. 4) has been detected in ESH.^42^

## 22. Sargassum kjellmanianum

(+)-Kjellmanianone 49 (Fig. 4), is a highly oxygenated cyclopentenone isolated from *S. kjellmanianum* collected from the Bay of Hiroshima (Japan), and was shown to have moderate antibacterial activity against *E. coli* and *B. subtilis* var. niger, Gram-positive microorganisms.^43^ Sargassumlactam 50 (Fig. 4) was also detected in *S. kjellmanianum* and was shown to have a moderate antibacterial effect against *S. aureus* and *E. coli*.^44^ An aqueous extract of *S. kjellmanianum* collected from Hahakimoku (Japan) was effective in the *in vivo* growth inhibition of implanted Sarcoma-180 cells, but showed no effect against L-1210-bearing mice. Polysaccharide fractions containing l-fucose and ester sulfate in the amounts of 12.6%, and 15.4%, 23.5%, and 17.2%, respectively, were extracted from *S. kjellmanianum*. The sulfated polysaccharide was effective against L-1210 leukemia with an ILS value of 26%.^45^ The *in vitro* immune-regulatory and anti-tumor activities of sulfated polysaccharides from a hot-water crude extract of *S. kjellmanianum* (SKP) were detected; the polysaccharides were composed of l-fucose, d-galactose, d-xylose, and d-mannose. All three types of polysaccharides were sulfated, with the sulfate group accounting for 3.4%, 25.6%, and 29.9% of SKP1, SKP2, and SKP3, respectively. SKP2 and SKP3 significantly enhanced the immune function of immunocytes, and SKP2 increased the proliferation rate of spleen lymphocytes and peritoneal macrophages by up to 53.56% and 51.41%. Cell culture showed that SKP1, SKP2, and SKP3 all inhibited the proliferation of HT-29 and HeLa cells.^46^

## 23. Sargassum ilicifolium

The anticonvulsant activity of *S. ilicifolium* collected from Bhatkarwada (India) in maximal electroshock (MES)- and pentylenetetrazole (PTZ)-induced convulsion in mice was detected. Chloroform (600 mg kg^−1^) and ethanol extracts (400 mg kg^−1^ and 600 mg kg^−1^) of *S. ilicifolium* significantly downregulated the duration of tonic hind limb extension in a maximal electroshock (MES) model and upregulated the latency to the onset of convulsions in a pentylenetetrazol (PTZ) model. These results were comparatively similar to the effects of phenytoin (25 mg kg^−1^) and phenobarbitone (20 mg kg^−1^).^47^

## 24. Sargassum integerrimum

Heteropolysaccharides were detected in *S. integerrimum*, and were shown to have neuroprotective and antioxidant activities.^48^

## 25. Sargassum lacerifolium

Dimethylarsonioboside 51, [deoxy(dimethylarsinoyl)ribosyl]mannitol 52, and methyl 5-deoxy(dimethylarsinoyl) riboside 53 (Fig. 4) are arsenic-containing ribosides, isolated from the methanol extract of *S. lacerifolium* collected from Mullaloo Beach (Australia).^49^

## 26. Sargassum latifolium

Polysaccharides (E1–E4) were detected in *S. latifolium* collected from in the Red Sea (Egypt). E1 and E4 were potent anti-initiators, where they lead not only to an inhibition in the carcinogen activator cytochrome P450 1A (IC_50_ 2.54 and 10.30 μg mL^−1^, respectively) but also to an induction in the carcinogen detoxification enzymes glutathione-*S*-transferases (144% and 225% of the control, respectively). E1 and E4 inhibited 59% and 63% of the induced-DNA damage. The anti-inflammatory activity of E1 and E4 enhanced the macrophage proliferation, inhibited the stimulated NO (30.7% and 59.3%), TNF-α (38.2% and 54.9), and COX-2 (20% and 18%), respectively. E3 showed also a selective cytotoxicity against lymphoblastic leukemia (1301 cells).^50^

## 27. Sargassum linifolium

The carbohydrate moiety of sargassan involves a backbone chain of d-glucuronic acid and d-mannose residues, and side chains involving residues of d-galactose, d-xylose, and l-fucose with sulfate attached to some galactose and fucose residues were detected in *S. linifolium* collected from Alexandria (Egypt).^51^

## 28. Sargassum macrocarpum

Sargafuran 54 (Fig. 4) was detected in a methanol extract of *S. macrocarpum*, collected from north to south along the Japanese coastline, and showed activity against *Propionibacterium acnes*, with an MIC of 15 μg mL^−1^.^52^ Sargachromenol 55, isolated from *S. macrocarpum*, had a marked nerve growth factor (NGF)-dependent neurite outgrowth promoting activity to PC12D cells with a median effective dose (ED_50_) of 9 μM, against PC12D cells in the presence of 10 ng mL^−1^ NGF. Sargachromenol 55 significantly promoted the survival of neuronal PC12D cells at 0–50 ng mL^−1^ NGF in a serum-free medium.^53^ Tuberatolide B 56 (Fig. 4) is a diastereomeric meroterpenoid detected in *S. macrocarpum*, and inhibits tumor growth in breast, lung, colon, prostate, and cervical cancer cells, and suppresses cancer progression by promoting the ROS-mediated inhibition of STAT3 signaling.^54^ The anti-inflammatory effects of an extract of *S. macrocarpum* (SME), collected from Jeju Island (Korea), in bone marrow-derived macrophages (BMDMs) and dendritic cells (BMDCs) were detected. Primary BMDMs and BMDCs were used for cytokine production and western blot analysis. SME (0–50 μg mL^−1^) pre-treatment led to a strong inhibitory effect against IL-12 p40, IL-6, and TNF-α, production in CpG-stimulated BMDMs and BMDCs. SME pre-treatment caused a strong inhibitory effect against NF-κB activation.^55^

## 29. Sargassum mangarevense

*S. mangarevense* collected from Tahiti contained alginate (9.3% ± 1.7% dw, M/G = 1.42 ± 0.24), mannitol (12.2% ± 2.1% dw), and phenolic contents (2.85% ± 1.12% dw). The aqueous and ethanol extracts of *S. mangarevense* showed antimicrobial activity against *S. aureus* with MICs of 9.5 and 12.5 mm, respectively.^56^

## 30. Sargassum marginatum

The growth inhibition of human pro-myelocytic leukemia (HL-60) cells by lipid extracts of *S. marginatum* collected from Goa (west coast of India) was detected with special reference to the fatty acid composition. PL exhibited cytotoxic activity at concentrations <20 μg mL^−1^.^57^

## 31. Sargassum micracanthum

**Fig. 5:**
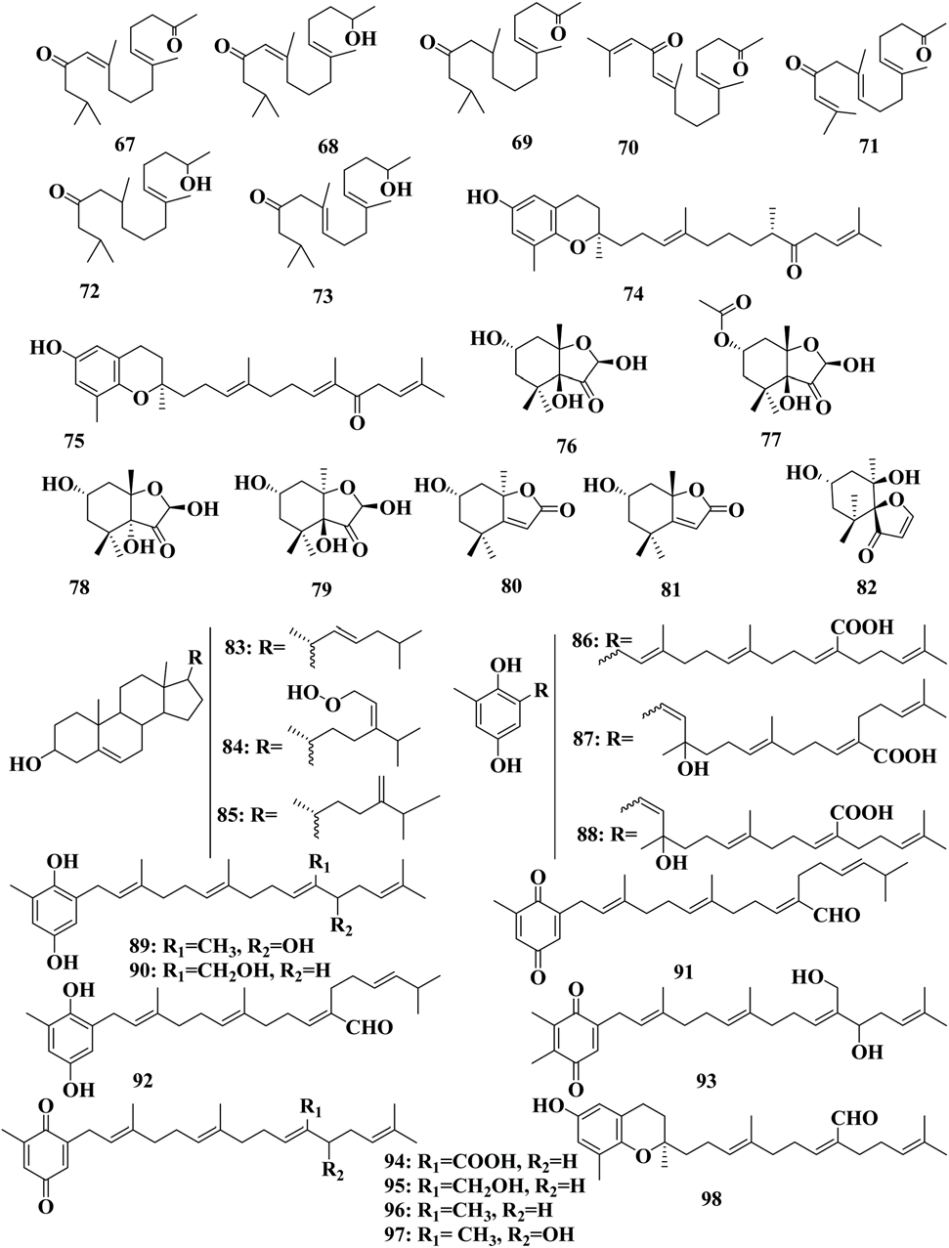
Chemical structures of compounds 67–97.

Fucosterol 5, sargachromenol 55, and sargaquinoic acid 57 were detected in *S. micracanthum* collected on Jeju Island (Korea). Sargachromenol 55 and sargaquinoic acid 57 were found to scavenge DPPH (IC_50_ = 49.3 and 100.2 μM).^58^ 2-[(2*E*,6*E*,10*E*)-15-Hydroxy-3,7,11,15-tetramethyl-14-oxohexadeca-2,6,10-trien-1-yl]-6-methylcyclohexa-2,5-diene-1,4-dione 58, (6*E*,10*E*,14*E*)-16-(2,5-dihydroxy-3-methylphenyl)-2-hydroxy-2,6,10,14-tetramethylhexadeca-6,10,14-trien-3-one 59, 3-[(2*E*,6*E*,10*E*,14*R*)-14,15-dihydroxy-3,7,11,15-tetramethylhexadeca-2,6,10-trien-1-yl]-4-hydroxy-5-methylphenyl octadecanoate 60, 3-[(2*E*,6*E*,10*E*,14*R*)-14,15-dihydroxy-3,7,11,15-tetramethylhexadeca-2,6,10-trien-1-yl]-4-hydroxy-5-methylphenyl(9*Z*)-octadec-9-enoate 61, and 2-geranylgeranyl-6-methylhydroquinone 62 are plastoquinones detected in a methanol extract of *S. micracanthum* collected from the Toyama Bay coast (Japan). Compounds 59–61 have a reductive effect on DPPH (3.00%, 52.6%, and 32.3%, respectively), and cytotoxicity against colon 26-L5 (IC_50_ 1.51, 17.5, and 1.69 μg mL^−1^, respectively) at a dose of 100 mg mL^−1^ of the sample.^59^ 2-Geranylgeranyl-6-methylhydroquinone 62 and 2-geranylgeranyl-6-methylbenzoquinone 63 were detected also in a methanol extract of *S. micracanthum* collected from the Toyama Bay (Japan). Compounds 62 and 63 showed an inhibitory effect on lipid peroxidation with IC_50_ = 0.11 and 1.0 μg mL^−1^, respectively. While their antiviral effects were detected against HSV-1, HSV-2, HCMV, mumps virus, measles virus, adeno virus, influenza virus, polio virus, and coxsackie virus, with IC_50_ ranging from 7.7 to 35 μM.^60^ Sargassumol 64 was detected in a methanol extract of *S. micracanthum* collected from Wando County (Korea). Sargassumol 64 showed minimal scavenging activity against DPPH radical, but exhibited the potent ABTS radical-scavenging activity with an IC_50_ of 47 μM, comparable to that of trolox (IC_50_ 45 μM).^61^ Dihydromonooxofranesylacetone 65 and Δ10-(11)-dihydromonooxofranesylacetone 66 were detected in an acetone extract of *S. micracanthum* collected from the coast of Gosa (Japan) (Fig. 4).^62^ Dihydromonooxofarnesylacetone 67, (5*Z*,10*E*)-14-hydroxy-2,6,10-trimethylpentadeca-5,10-dien-4-one 68, (5*E*)-6,10,14-trimethylpentadec-5-ene-2,12-dione 69, (5*E*,10*E*)-6,10,14-trimethylpentadeca-5,10,13-triene-2,12-dione 70, (5*E*,9*E*)-6,10,14-trimethylpentadeca-5,9,13-triene-2,12-dione 71, (10*E*)-14-hydroxy-2,6,10-trimethylpentadec-10-en-4-one 72, and (6*E*,10*E*)-14-hydroxy-2,6,10-trimethylpentadeca-6,10-dien-4-one 73 are farnesylacetone derivatives isolated from a methanol extract of Japanese *S. micracanthum*.^63^ (2*R*,8′*S*)-7′,8′-Dihydro-9′-oxo-δ-tocotrienol 74, and (2*R*)-9′-oxo-δ-tocotrienol 75 were detected in a methanol extract of *S. micracanthum*, collected from Toyama Bay (Japan) (Fig. 5).^64^

## 32. Sargassum muticum

1-Dodecene, 1-tetradecene, 1-pentadecene, *n*-pentadecene, 1-hexadecene, *n*-heptadecane, 1-octadecene, (*E*)-3-eicosene, *n*-tricosane, *n*-tetracosane, *n*-pentacosane, *n*-hexacosane, and *n*-heptacosane hydrocarbons, myristic acid, pentadecanoic acid, palmitic acid 7, stearic, palmitoleic, oleic, vaccenic acid, hexadecadienoic acid, linolenic, stearidonic, arachidonic, and eicosapentaenoic fatty acids were detected in GC-MS of a chloroform extract of *S. muticum* collected from Plouzané (France).^65^ Saringosterol 11 was detected in *S. muticum*, which is widely distributed in Korea, and exhibited an anti-obesity effect. The inhibitory effect of saringosterol 11 on adipogenesis was detected *via* Oil Red O staining in 3T3-L1 preadipocytes in a dose-dependent manner, which inhibited adipocyte differentiation and expression of adipogenic marker genes.^66^

## 33. Sargassum myriocystum

Sulfated polysaccharides extracted from *S. myriocystum* (SMP) were investigated for gentamicin-induced nephrotoxicity in adult zebrafish. The SMP showed maximum carbohydrate, sulfate and l-fucose contents, which suppressed mRNA expression levels of KIM-1, NF-κB, TNF-α, and IL-6 in a dose-dependent manner.^67^

## 34. Sargassum naozhouense

Glutamic acid (13.21 g/100 g protein), aspartic acid (8.39 g/100 g protein), alanine(5.27 g/100 g protein), glycine (4.38 g/100 g protein), tyrosine, threonine, phenylalanine, serine, histidine, lysine, proline, arginine, tryptophan, valine, methionine, cysteine, isoleucine, and leucine comprised the amino acid composition of cultivated *S. naozhouense*, collected from Techeng Island (China), while the main fatty acids in the cultivated *S. naozhouense* were myristic acid, palmitic acid, oleic acid, and arachidonic acid. Water-soluble polysaccharides were detected in an extract of *S. naozhouense*, which contained sulfate groups, and exhibited strong antiviral activity against HSV-1 strain, with an IC_50_ of 8.92 μg mL^−1^.^68^ Sargassumone 76, (2*R*,6*S*,8*S*,9*S*)-hexahydro-2,9-dihydroxy-4,4,8-trimethyl-6-acetyloxy-3(2*H*)-benzofuranone 77, (6*S*,8*S*,9*R*)-hexahydro-6,9-dihydroxy-4,4,8-trimethyl-2(2*H*)-benzofuranone 78, (6*S*,8*S*,9*R*)-hexahydro-6,9-dihydroxy-4,4,8-trimethyl-2(2*H*)-benzofuranone 79, loliolide 80, (+)-epiloliolide 81, and spheciospongones A 82 as norisoprenoid derivatives and a highly oxygenated cyclopentene were detected in *S. naozhouense* (Fig. 5).^69^

## 35. Sargassum natans

Sodium alginate was detected in *S. natans* collected from both Manzanilla and Mayaro Bays (Trinidad).^70^

## 36. Sargassum oligocystum

Fucosterol 5, cholesterol 6, 24-hydroperoxy-24-vinylcholesterol 24, a mixture of 24(*S*)-hydroxy-24-vinylcholesterol 38, and 24(*R*)-hydroxy-24-vinylcholesterol 39, 22-dehydrocholesterol 83, 29-hydroperoxystigmasta-5,24(28)-dien-3β-ol 84, and ostreasterol 85 were detected in *S. oligocystum* collected from the Persian Gulf (Fig. 5). A chloroform extract of *S. oligocystum* was moderately effective against *A. salina* nauplii (LC_50_ = 159 μg mL^−1^).^71^ A water extract of *S. oligocystum* collected from the Persian Gulf was detected to have antitumor activity against K562 and Daudi cell lines at concentrations of 500 μg mL^−1^, and 400 μg mL^−1^, respectively.^72^

## 37. Sargassum pallidum

An ethanol crude extract of *S. pallidum* collected from Weihai (China) showed antioxidant activity against DPPH.^73^

## 38. Sargassum patens

The anti-inflammatory effect of an ethanol extract of *S. patens* (SPEE) collected from Busan (Korea) was detected. The production of NO was suppressed by SPEE to 28%, at 100 μg mL^−1^, and the levels of IL-6, TNF-α, and IL-1β were dose-dependently suppressed. *In vivo*, a croton oil-induced mouse ear edema was attenuated by SPEE and the infiltration of mast cells into the tissue was suppressed.^74^ A sulfated polysaccharide was detected in *S. patens*, and was reported to inhibit the replication of herpes simplex virus type 2 (HSV-2) in a dose-dependent manner by 38.5–96.1%, of the control level, after incubation with 0.78–12.5 μg mL^−1^ of the polysaccharide. The sulfated polysaccharide exhibited extracellular virucidal activity only in high concentrations (≥12.5 μg mL^−1^), but suppressed the virus attachment to its host cells by 45.1%, at a concentration < 1 μg mL^−1^.^75^

## 39. Sargassum paradoxum

Fallahydroquinone 30, fallaquinone 31, sargahydroquinoic acid 86, chabrolohydroxybenzaquinone A 87, chabrolohydroxybenzaquinone C 88, paradoxhydroquinone 89, 2-[11-(hydroxymethyl)-3,7,15-trimethyl-2,6,10,14-hexadecatetraen-1-yl]-6-methyl-1,4-benzenediol 90, sargaquinal 91, sargahydroquinal 92, paradoxquinol 93, sargahydroquinoic acid 94, 2-[11-(hydroxymethyl)-3,7,15-trimethyl-2,6,10,14-hexadecatetraen-1-yl]-6-methyl-1,4-benzoquinone, 95, sargaquinone 96, paradoxquinone 97, and (3′*E*,7′*Z*)-9-(6-hydroxy-2,8-dimethyl-2*H*-1-benzopyran-2-yl)-6-methyl-2-(4-methyl-3penten-1-yl)-2,6-nonadienal 98 were detected in a crude extract of *S. paradoxum* collected from Port Phillip (Australia) (Fig. 5).^76^

## 40. Sargassum parvivesiculosum

**Fig. 6:**
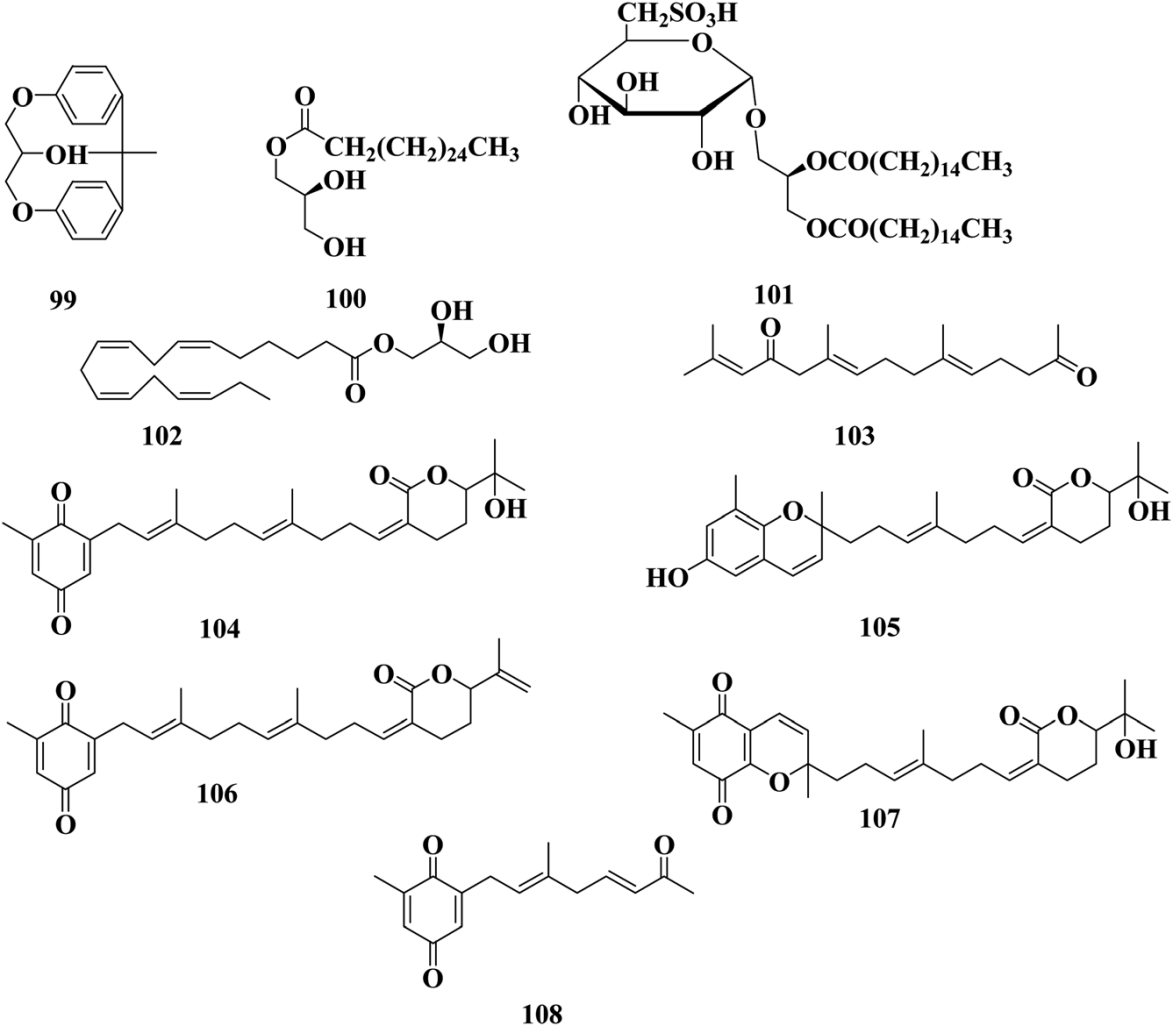
Chemical structures of compounds 99–108.

1,3-Di-*O*-[2′,2′-di-(*p*-phenylene) isopropylidene]glycerol 99, (2*S*)-1-*O*-heptatriacontanoyl glycerol 100, and (2*S*)-1,2-di-*O*-palmitoyl-3-*O*-(6-sulfo-α-d-quinovopyranosyl)glycerol 101 are glycerol derivatives isolated from an ethanol extract of *S. parvivesiculosum* collected from Sanya (China) (Fig. 6).^77^

## 41. Sargassum polycystum

The total yield of fucoidan isolated from *S. polycystum* was 4.51 ± 0.24%, and contained 46.8% of l-fucose and 22.35% ± 0.23% of sulfate respectively. The cytotoxicity effect of fucoidan, showed a higher percentage (90.4% ± 0.25%) of inhibition against the MCF-7 cell line, at 150 μg mL^−1^, with an IC_50_ of 50 μg mL^−1^.^78^

## 42. Sargassum ringgoldianum

An extract *of S. ringgoldianum* collected from Jeju Island (Korea), showed the inhibition of α-glucosidase and α-amylase, and alleviated postprandial hyperglycemia in streptozotocin-induced diabetic mice. The IC_50_ values of *S. ringgoldianum* extract against α-glucosidase and α-amylase were 0.12, and 0.18 mg mL^−1^, respectively. The blood glucose levels of the *S. ringgoldianum* extract in the administered group were significantly lower in the streptozotocin-induced diabetic mice compared to in the control group.^79^

## 43. Sargassum sagamianum

An ethanol extract of *S. sagamianum* collected from Yeonhwari (Korea) was detected to include an anti-inflammatory agent, whereby the rate of formation of edema in a mouse ear model was reduced by 46%, using a dose of 250 mg kg^−1^. The ethanol extract showed potent inhibitory effects on the LPS-induced expression of inflammatory mediators, such as NO, iNOS, COX-2, and cytokines, in macrophages through suppression of the NF-κB p65 pathway.^80^ An ethanol extract of *S. sagamianum* collected from Jeju Island (Korea) was investigated on INS-1 pancreatic β cells against high glucose-induced oxidative stress and apoptosis at high concentrations (30 mM) causing β cell apoptosis, whereby treatment with SSE protected the β cells from high glucose-induced damage by recovering the cell viability. Treatment with the ethanol extract at concentrations of 10–100 μg mL^−1^ decreased lipid peroxidation, intracellular ROS, and nitric oxide levels, as well as increased cell viability and insulin secretion in high glucose pre-treated INS-1 cells, in a dose-dependent manner.^81^ 1-Octadecatetraenoyl glycerol 102 is a polyunsaturated fatty acid-derived monoglyceride detected in *S. sagamianum* collected from Jeju Island (Korea). A series of monoglycerides were synthesized using glycerol and various fatty acids. Several synthesized compounds showed a moderate to significant inhibition of phospholipase A2 and cyclooxygenase-2.^82^ Sargachromenol 55, sargaquinoic acid 57, dihydromonofarnesylacetone 65, and monooxofarnesylacetone 103 were isolated from a methanol extract of *S. sagamianum*. These compounds showed moderate acetylcholinesterase (AChE) inhibitory activity in a micromole range.^83^*In vitro*, treatment with sargachromenol 55 and sargaquinoic acid 57 promoted cell death and the activation of caspase-3, caspase-8, caspase-9, and poly (ADP-ribose) polymerase (PARP) in a concentration-dependent manner. Sargaquinoic acid- or sargachromenol-induced apoptosis was enhanced by co-treatment with UVB irradiation. The topical application of sargaquinoic acid (1 mg mL^−1^) before UVB irradiation (2.5 kJ m^−2^) to hairless mice also enhanced apoptosis, including the activation of caspase-3.^84^ 15′-Hydroxysargaquinolide 104, 11′-hydroxysargachromelide 105, 15′-methylenesargaquinolide 106, chromequinolide 107, and (2′*E*,5′*E*)-2-methyl-6-(7′-oxo-3′-methylocta-2′,5′-dienyl)-1,4-benzoquinone 108 were detected in *S. sagamianum* collected from Manazuru in the Kanagawa prefecture. Compounds 104, 105, and 108, had antibacterial activity against *S. aureus*, (MIC 16, 128, and 2 μg mL^−1^, respectively) (Fig. 6).^85^

## 44. Sargassum serratifolium

An ethanol extract of *S. serratifolium* collected from Busan (Korea) ameliorated paw swelling, reduced the arthritis score, decreased the secretion of pro-inflammatory cytokines in the serum and joint tissue, and suppressed the collagen-induced rheumatoid arthritis of mice. The ethanol extract showed a downregulated NF-κB signaling pathway by suppressing the phosphorylation of protein kinase B, c-Jun N-terminal kinase, and p38 mitogen-activated protein kinases.^86^ An ethanol extract of *S. serratifolium* exhibited potential antimicrobial activity against pathogenic commensal bacteria related to *acne vulgaris* (*P. acnes*, *S. epidermidis*, *S*. *aureus*, and *P. aeruginosa*), and *C*. *albicans*, which causes cutaneous candidiasis. Among the solvent-soluble fractions from the ethanol extract, the *n*-hexane fraction showed the strongest antimicrobial activity against all the tested human skin pathogens (MIC from 32 to 512 μg mL^−1^).^87^ An ethanol extract of *S. serratifolium*, collected from Tongyoung (Korea), which mainly contained sargachromenol 55 and sargaquinoic acid 57, efficiently suppressed adipocyte differentiation and lipid accumulation in 3T3-L1 cells by downregulating the proteins involved in cell cycle progression and adipogenesis, and also downregulated the key transcription factors, such as C/EBPβ, C/EBPα, PPARγ, RXRα, SREBP1c, and STAT3, which were responsible for the observed suppression of lipid accumulation upon treatment with an ethanol extract of *S. serratifolium*.^88^

## 45. Sargassum siliquastrum

**Fig. 7:**
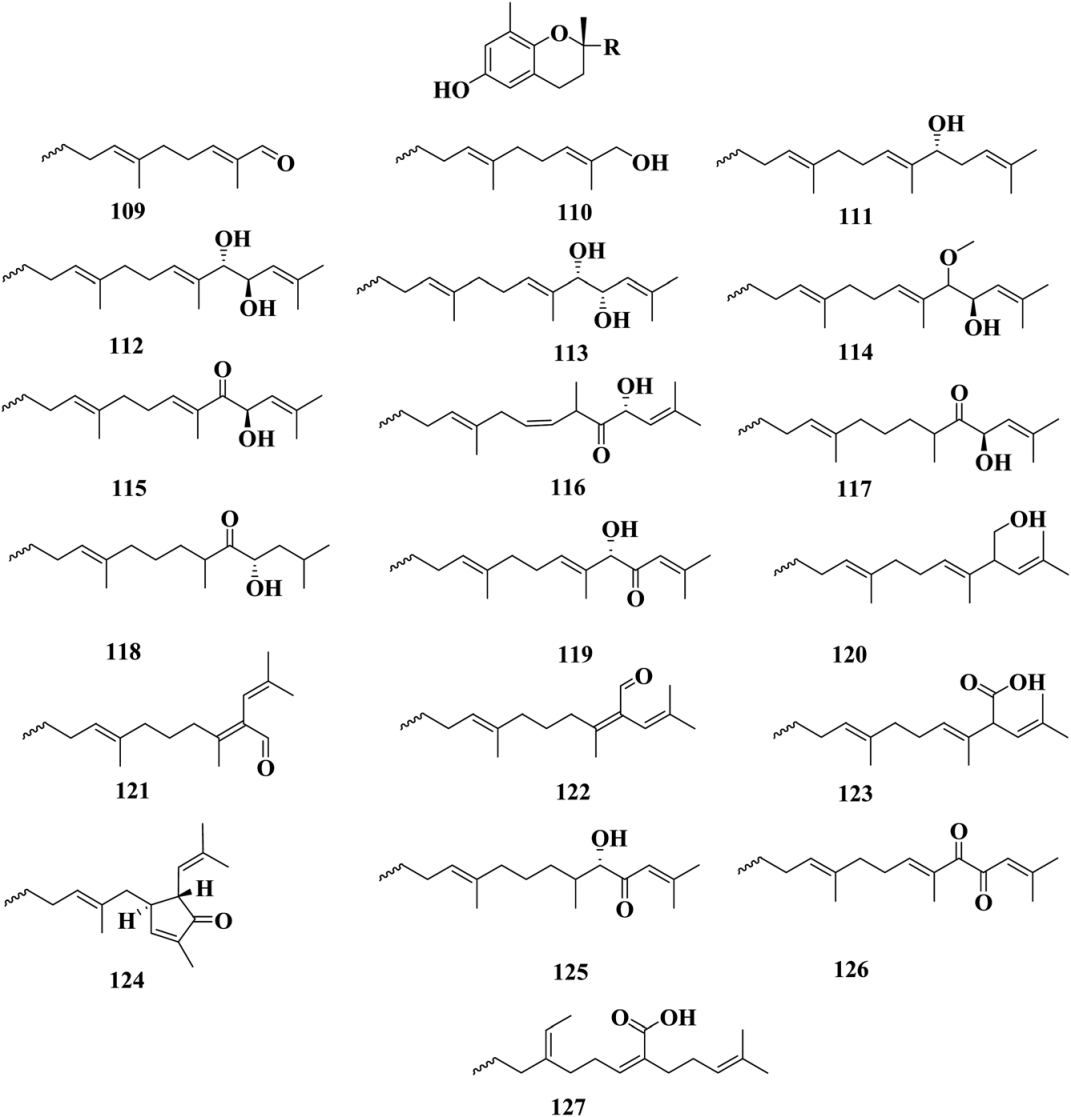
Chemical structures of compounds 109–127.

**Fig. 8:**
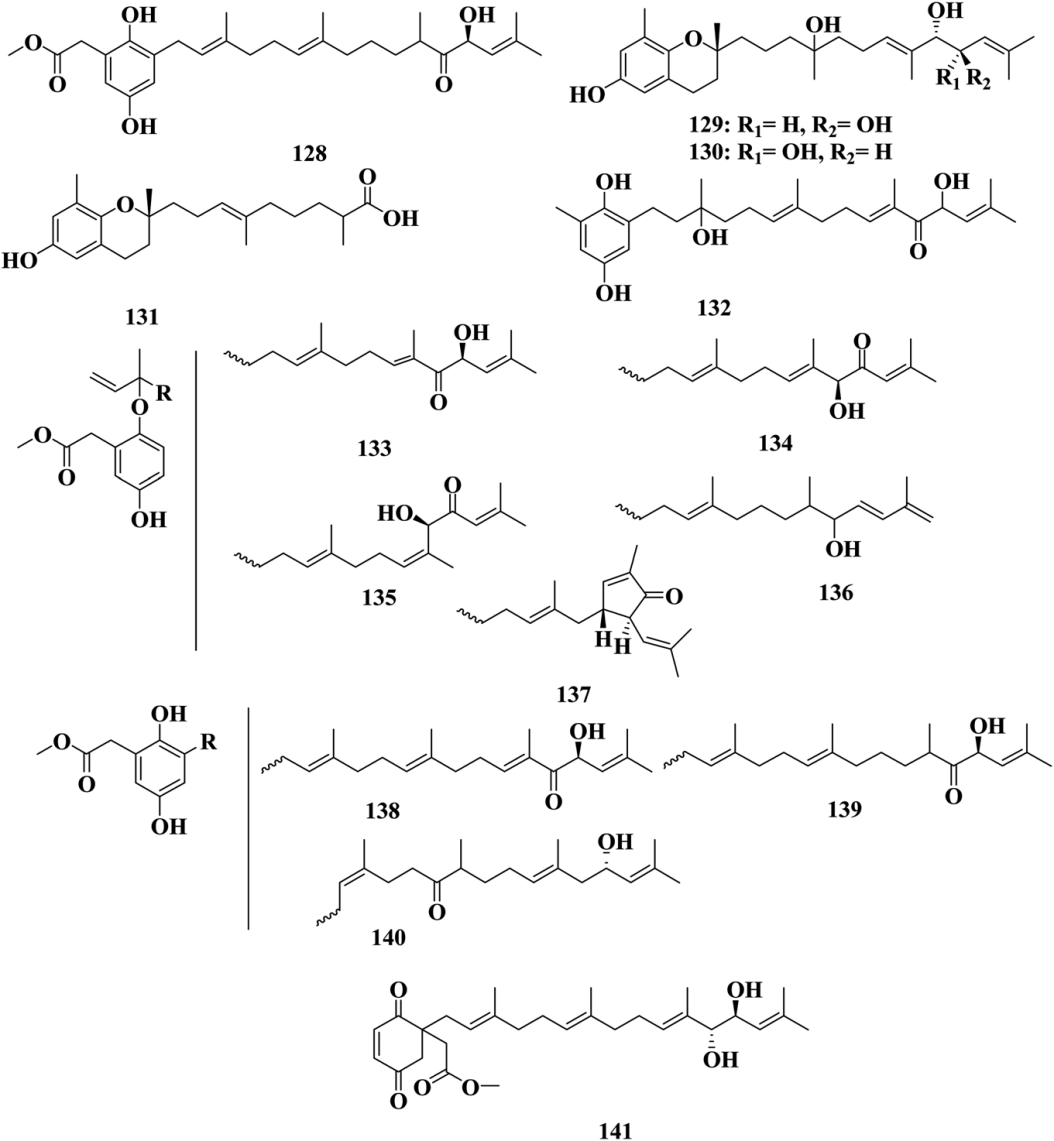
Chemical structures of compounds 128–141.

Sargachromanol A–P 109–124 are meroterpenoids detected in *S*. *siliquastrum*. These compounds exhibited significant antioxidant activity in the DPPH assay. Compounds 115 and 123 also showed inhibitory activity toward butylcholine esterase.^89^ Sargachromanol Q 125 and sargachromanol R 126 were detected in *S. siliquastrum* collected from Cheju Island (Korea). Sargachromanol R 126 had potent cytotoxic activity against AGS, HT-29, and HT-1080 cell lines, with IC_50_ values of 6.5, 3.4, and 13.9 μg mL^−1^, respectively, compared with paclitaxel and doxorubicin.^90^ Mojabanchromanol 127 was detected in an ethanol extract of *S. siliquastrum* collected from the seashore of Pusan (Fig. 7).^91^ Isonahocol E3 128, detected in *S*. *siliquastrum*, has functional antagonistic activities against ET-1 induced inflammatory and pro-angiogenic effects, through inhibition of ET-1-induced cell proliferation, as well as inflammatory mediators, like IL-6, IL-8, and TNF-α, and pro-angiogenic factors, including metalloproteinases in immortalized human keratinocytes. Isonahocol E3 128 also reduced the expression level of endothelin ETA receptor and endothelin ETB receptor, as well as suppressed ET-1-induced extracellular signal-regulated kinase (ERK) phosphorylation.^92^ Sargachromanol D 112, sargachromanol E 113, sargachromanol K 119, sargachromanol P 124, (9*S*,10*S*)-13-(3,4-dihydro-6-hydroxy-2,8-dimethy-2*H*-1-benzopyran-2-yl)-2,6,10-trimethyl-trideca-(2*E*,6*E*)-diene-4,5,10-triol 129, (9*S*,10*R*)-13-(3,4-dihydro-6-hydroxy-2,8-dimethy-2*H*-1-benzo-pyran-2-yl)-2,6,10-trimethyl-trideca-(2*E*,6*E*)-diene-4,5,10-triol 130, and 9-(3,4-dihydro-6-hydroxy-2,8-dimethy-2*H*-1-benzopyran-2-yl)-2,6-dimethyl-(6*E*)-nonenoic acid 131 were detected in *S. siliquastrum* collected from JeJu Island (Korea). Sargachromanols A 109, B 110, and K 119, had strong cytotoxicity against AGS, HT-29, HT-1080, and MCF-7 cell lines, with IC_50_ values of 0.7, 6.1, 0.7, and 28.1 μg mL; 0.5, 1.0, 3.3, and 23.6 μg mL^−1^ and 5.7, 0.8, 1.8, and 10.3 μg mL^−1^, respectively, comparable with paclitaxel and doxorubicin.^93^ (6*E*,10*E*)-16-(2,5-Dihydroxy-3-methylphenyl)-4,14-dihydroxy-2,6,10,14-tetramethylhexadeca-2,6,10-trien-5-one 132, methyl(5-hydroxy-2-{[(6*E*,10*E*,13*S*)-13-hydroxy-3,7,11,15-tetramethyl-12-oxohexadeca-1,6,10,14-tetraen-3-yl]oxy}phenyl)acetate 133, methyl(5-hydroxy-2-{[(6*E*,10*E*,12*S*)-12-hydroxy-3,7,11,15-tetramethyl-13-oxohexadeca-1,6,10,14-tetraen-3-yl]oxy}phenyl)acetate 134, methyl(5-hydroxy-2-{[(6*E*,10*Z*,12*R*)-12-hydroxy-3,7,11,15-tetramethyl-13-oxohexadeca-1,6,10,14-tetraen-3-yl]oxy}phenyl)acetate 135, methyl(5-hydroxy-2-{[(6*E*,13*E*)-12-hydroxy-3,7,11,15-tetramethylhexadeca-1,6,13,15-tetraen-3-yl]oxy}phenyl)acetate 136, methyl[2-({(6*E*)-3,7-dimethyl-8-[(1*R*,5*R*)-3-methyl-5-(2-methylprop-1-en-1-yl)-4-oxocyclopent-2-en-1-yl]octa-1,6-dien-3-yl}oxy)-5-hydroxyphenyl]acetate 137, methyl{2,5-dihydroxy-3-[(2*E*,6*E*,10*E*,13*S*)-13-hydroxy-3,7,11,15-tetramethyl-12-oxohexadeca-2,6,10,14-tetraen-1-yl]phenyl}acetate 138, methyl{2,5-dihydroxy-3-[(2*E*,6*E*,13*S*)-13-hydroxy-3,7,11,15-tetramethyl-12-oxohexadeca-2,6,14-trien-1-yl]phenyl}acetate 139, methyl{2,5-dihydroxy-3-[(2*Z*,10*E*,13*S*)-13-hydroxy-3,7,11,15-tetramethyl-6-oxohexadeca-2,10,14-trien-1-yl]phenyl}acetate 140 and methyl{1-[(2*E*,6*E*,10*E*,12*R*,13*S*)-12,13-dihydroxy-3,7,11,15-tetramethylhexadeca-2,6,10,14-tetraen-1-yl]-2,5-dioxocyclohex-3-en-1-yl}acetate 141 were detected in *S*. *siliquastrum*. Compounds 132–141 were revealed to have a common tetraprenyl hydroquinone structure, which belonged to the nahocol, isonahocol, and sargahydroquinoic acid classes. The dihydroquinone moiety of 141 was unique and unprecedented in a brown alga. These compounds exhibited moderate to significant radical-scavenging activity as well as weak inhibitory activities against sortase A, and isocitrate lyase (Fig. 8).^94^

## 46. Sargassum siliquosum

Methanol extracts of *S. siliquosum* (ME) and a fucoxanthin-rich fraction (FRF) showed antioxidant activity, with IC_50_ values of 0.2, and 0.04 mg mL^−1^, respectively. Also, the IC_50_ values for the angiotensin-converting enzyme (ACE) inhibitory activity of ME (IC_50_ = 0.03–0.42 mg mL^−1^) was lower than that of FRF (0.94–1.53 mg mL^−1^). CE and FRF also showed α-glucosidase inhibitory activity, with IC_50_ values of 0.57, and 0.50 mg mL^−1^, respectively.^95^

## 47. Sargassum spinuligerum

Deshydroxytetrafuhalol-A, tetrafuhalol-A, pentafuhalol-A, hexafuhalol-A, heptafuhalol-A, octafuhalol-A, nonafuhalol-A and undecafuhalol-A, the acetylated derivatives of deshydroxytetrafuhalol-C, deshydroxyhexafuhalol-A, deshydroxyhexafuhalol-C and deshydroxyhexafuhalol-D, deshydroxyoctafuhalol-C, octafuhalol-B, decafuhalol-A, dodecafuhalol-A, tetradecafuhalol-A, hexadecafuhalol-A, octadecafuhalol-A and eicosafuhalol-A were the largest phlorotannins found in an ethanol extract of *S*. *spinuligerum*, collected from Whangaparoa Island, (Auckland).^96^ Phlorotannins, fucophlorethol-B octa-acetate, fucodiphlorethol-B, fucodiphlorethol-D and fucodiphlorethol-F deca-acetate, hydroxyfucodiphlorethol-A undeca-acetate, bisfucotriphlorethol-A pentadeca-acetate, hydroxybisfucophlorethol-A hexadeca-acetate, bisfucotetraphlorethol-A heptadeca-acetate, dihydroxyfucotriphlorethol-A and dihydroxyfucotriphlorethol-B tetradeca-acetate, bisfucopentaphlorethol-B nonadeca-acetate, chlorobisfucopentaphlorethol-A nonadeca-acetate, difucodiphlorethol-A trideca-acetate, fucodifucotetraphlorethol-A icosa-acetate, bisfucotriphlorethol-A pentadeca-acetate, chlorobisfucopentaphlorethol-A nonadeca-acetate, and fucodifucotetraphlorethol-A icosa-acetate were detected in *S. spinuligerum*.^97^ Pseudotrifuhalol-A, pseudotetrafuhalol-A, pseudopentafuhalols-A–D, pseudohexafuhols-A–C, pseudoheptafuhalols-A–D and psuedooctafuhalols-B–D were detected in an ethanolic extract of *S. spinuligerum*.^98^

## 48. Sargassum swartzii

Sulfated polysaccharide extracted (SPE) from *S. swartzii* was collected from a coastal region of Mandapam (India), with a molecular weight of 50 kDa, and with a high percentage of carbohydrate (7.40% ± 0.63%), followed by sulfate (5.3% ± 1.54%). The antioxidant activity of SPE detected for ABTS, H_2_O_2_ and DPPH, was 55% ± 3.61%, 47.23% ± 2.81%, and 25.33% ± 2.52%, respectively.^99^ Bioactive fucoidan fractions (CFF, FF1, and FF2) were detected in *S. swartzii* collected from the Gulf of Mannar (India), which contained mainly sugars, sulfate, and uronic acid. Fraction FF2 was found to exhibit significant anti-HIV-1 activity at concentrations of 1.56 μg mL^−1^ as observed by a >50% reduction in HIV-1 p24 antigen levels and reverse transcriptase activity.^100^

## 49. Sargassum tenerrimum

Sulfated polysaccharides, detected in *S. tenerrimum* collected from Mandapam (India), showed a higher percentage of carbohydrate (8.20% ± 1.23%) followed by sulfate (6.6% ± 1.42%) and protein (0.86% ± 0.42%). The free radical scavenging potential was found to be higher in ABTS (70.33% ± 2.33%) followed by DPPH (64.66% ± 2.08%) and H_2_O_2_ (61.56% ± 2.05%). The total antioxidant capacity (TAC) was found to be 62.55% ± 1.40%.^101^

## 50. Sargassum thunbergii

**Fig. 9:**
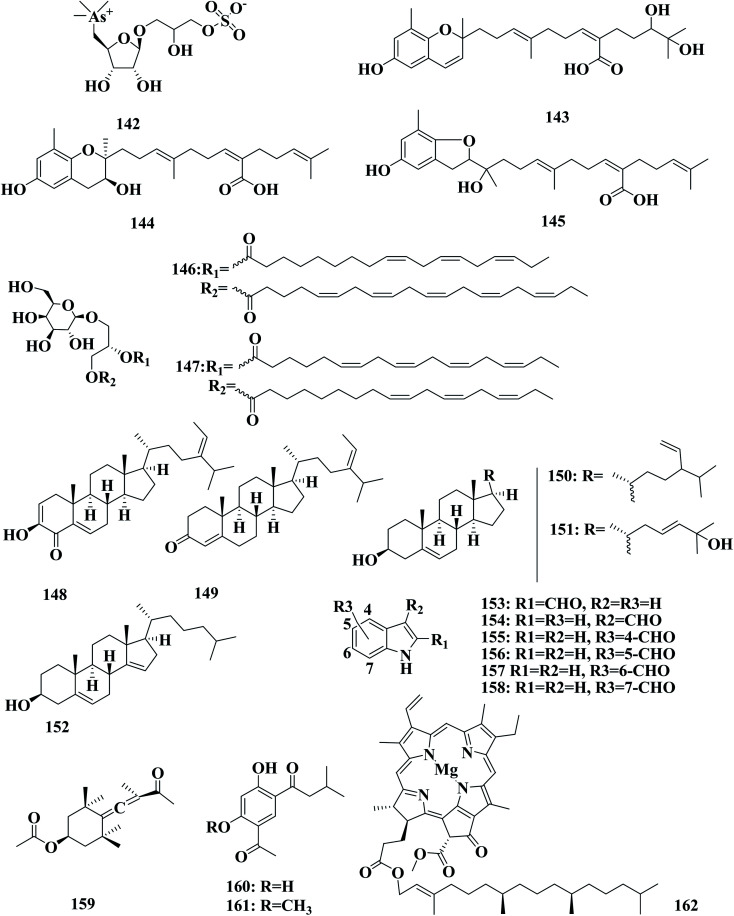
Chemical structures of compounds 142–162.

3-{[5-Deoxy-5-(trimethylarsonio) pentofuranosyl]oxy}-2-hydroxypropyl sulfate 142, arseno-sugar was detected in *S. thunbergii* collected from Nakaminato (Japan).^102^ Sargachrominol 55, sargaquinoic acid 57, sargahydroquinoic acid 94, and sargathunbergol A 143 were detected in *S. thunbergii* collected from Youngdo Island (Korea), which exhibited antioxidant activities (EC_50_ values of 32, 27,20, and 38 μg mL^−1^ respectively), compared to BHT (EC_50_ = 42 μg mL^−1^) and α-tocopherol (EC_50_ 23 μg mL^−1^).^103^ Sargahydroquinoic acid 95, sargaquinoic acid 2, and sargachromenol 55, thunbergol A 144, and thunbergol B 145, were detected in *S. thunbergii* collected from Busan (Korea). The evaluating capacity of compounds 144, and 145 to scavenge DPPH radical, showed they exhibited EC_50_ values of 30 and 31 μg mL^−1^, respectively.^104^

(2*S*)-1-*O*-(5*Z*,8*Z*,11*Z*,14*Z*,17*Z*-Eicosapentaenoyl)-2-*O*-(9*Z*,12*Z*,15*Z*-octadecatrienoyl)-3-*O*-β-d-galactopyranosyl-*sn*-glycerol 146 and (2*S*)-1-*O*-(9*Z*,12*Z*,15*Z*-octadecatrienoyl)-2-*O*-(6*Z*,9*Z*,12*Z*,15*Z*-octadecatetraenoyl)-3-*O*-β-d-galactopyranosyl-*sn*-glycerol 147 were glycolipids detected in the methanol extract of *S. thunbergii*, collected from the West Sea in Korea.^105^ Thunberol 148, along with 24-ethylcholesta-4,24(28)-dien-3-one 149, stigmasta-5,28-dien-3β-ol 150, cholesta-5,23-dien-3β,25-diol 151, and cholesta-5,14-dien-3β-ol 152, were detected in *S*. *thunbergii*, collected from Nanji Island (China). Thunberol 148 exhibited significant inhibitory activity against protein tyrosine phosphatase 1B, a potential compound target for the treatment of Type-II diabetes and obesity, with an IC_50_ value of 2.24 μg ml^−1^.^106^ (STCs)—indole-2-carboxaldehyde (STC-1) 153, indole-3-carboxaldehyde (STC-2) 154, indole-4-carboxaldehyde (STC-3) 155, indole-5-carboxaldehyde (STC-4) 156, indole-6-carboxaldehyde (STC-5) 157, and indole-7-carboxaldehyde (STC-6) 158 were detected in *S. thunbergii* and their inhibitory effects were evaluated on adipocyte differentiation in 3T3-L1 cells. STC-1 and STC-5 showed non-toxic inhibition of the differentiation of 3T3-L1 adipocytes. STC-1 and STC-5 significantly inhibited lipid accumulation and downregulated the expression of peroxisome proliferator-activated receptor-γ (PPARγ), CCAAT/enhancer-binding protein α (C/EBPα), and sterol regulatory element-binding protein 1c (SREBP-1c) in a dose-dependent manner. The specific mechanism mediating the effects of STC-1 and STC-5 was shown to be AMP-activated protein kinase (AMPK) activation. The inhibitory effect of STC-1, and STC-5 on adipogenesis was through activation of the AMPK signal pathway. STC-1 and STC-5 may be effective candidates for the prevention of obesity or obesity-related diseases.^107^ (+)-Epiloliolide 81, (−)-loliolide 80, and apo-9′-fucoxanthinone 159 norisoprenoids were detected in *S. thunbergii*, collected from Korea.^108^ 1-(5-Acetyl-2,4-dihydroxyphenyl)-3-methylbutan-1-one 160 and 1-(5-acetyl-2-hydroxy-4-methoxyphenyl)-3-methylbutan-1-one 161 were detected in a methanol extract of *S. thunbergii*, collected from Weihai City (China).^109^ Saringosterol 11, chlorophyll a 162, and isofucosterol 163, were also isolated from the chloroform fraction of *S. thunbergii* extract (Fig. 9).^110^

## 51. Sargassum trichophyllum

Laminaran, alginate, and fucoidan were detected in *S. trichophyllum*. Laminaran was found to be a β-glucan consisting of terminal- (14.5 mol%), 1,3- (69.4%), 1,6- (33.4%), and 1,3,6-linked (12.8%) residues. Alginate was found to be a mannuronic acid-rich alginate (M/G = 1.88). Fucoidan consisted of l-fucose (79.1 mol%) and d-galactose (19.9 mol%), and its sulfate content was estimated to be 25.5%, with antiviral activity against the herpes simplex virus type 2.^111^

## 52. Sargassum turbinarioides

Alginate was detected in *S. turbinarioides* collected from Nosy Bey (Madagascar), with a molecular weight of 5.528 × 10^5^ g mol^−1^.^112^

## 53. Sargassum vachellianum

**Fig. 10:**
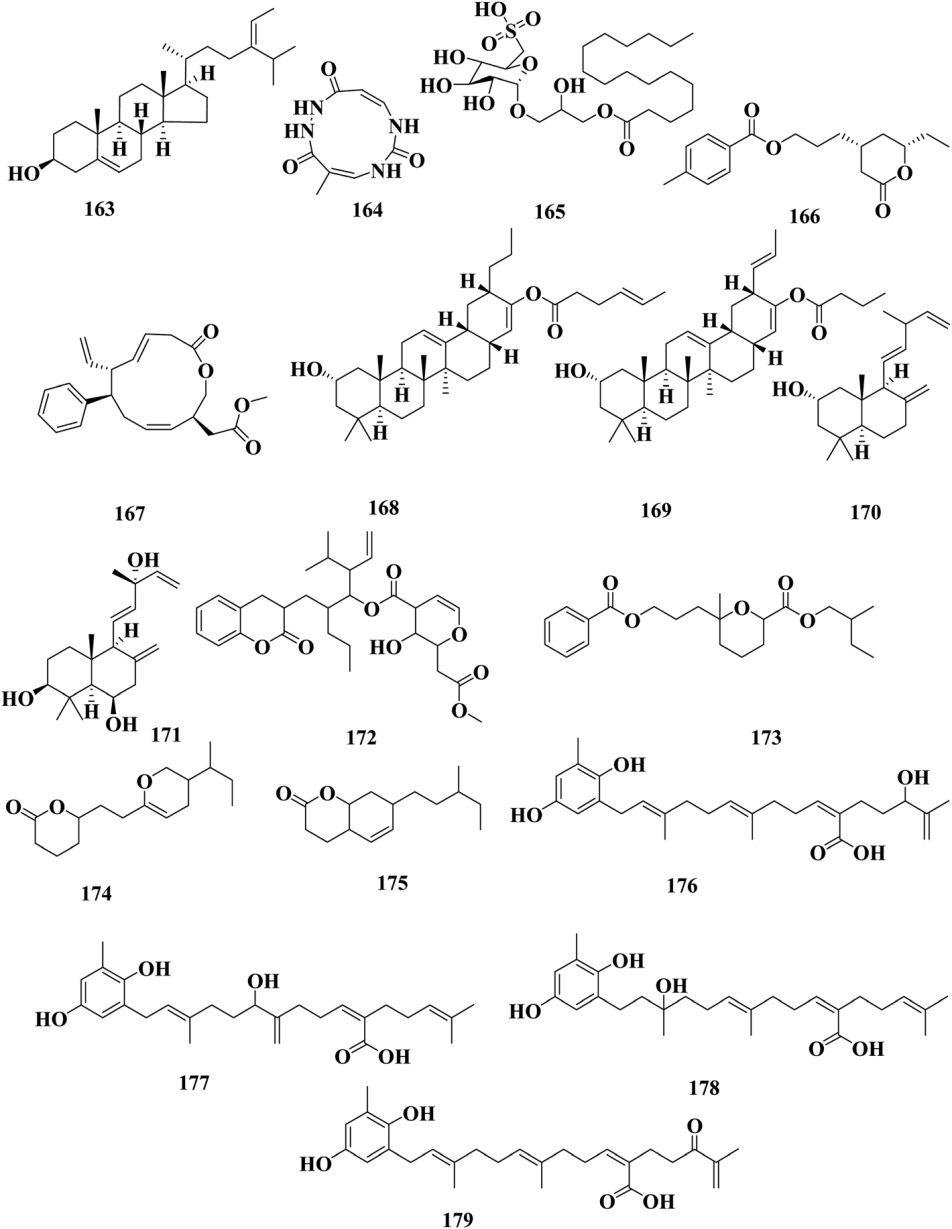
Chemical structures of compounds 163–179.

Fucoidan-rich polysaccharide extract (SPS) and polyphenol-rich extract (SPP) from *S. vachellianum*, collected from Nan'ao Island (China), showed a protective effect on the skin from UV damage. SPP showed good free radical scavenging ability, antimicrobial activity against *E. coli* and *S. aureus*, and effectively absorbed UVB and UVA rays. Whereas SPS hardly absorbs UVA and UVB rays and showed weak free radical scavenging ability and no antimicrobial activity. SPS showed considerable inhibition of tyrosinase (51.21%) and had better moisture absorption (52.1%) and retention (63.24%) abilities than SPP.^113^ (4*Z*,9*Z*)-4-Methyl-1,2,6,8-tetraazacycloundeca-4,9-diene-3,7,11-trione 164 is an eleven-membered macrocyclic hydrazide detected in *S. vachellianum* collected in the South China Sea (Fig. 10).^114^

## 54. Sargassum wightii

1-0 Palmitoyl-3-0(6′-sulfo-alpha-quinovopyranosyl)-glycerol 165, sulfoglycerolipid, was detected in a methanol extract of *S. wightii*, and showed antibacterial activity against *Xanthomonas oryzae* pv. *oryzae*, which causes bacterial blight of rice.^115^ 4-(8-Ethyl-tetrahydro-7-oxo-2*H*-pyran-5-yl)-propyl-4′-methylbenzoate 166 and methyl-2-(12-oxo-7-phenyl-8-vinyl-1-oxa-4,9-cyclododecadien-3-yl)-acetate 167, detected in the methanol extract of *S. wightii*, showed antioxidant activities against DPPH radical scavenging, with (IC_50_ values of 0.24–0.32 mg mL^−1^), compared to α-tocopherol (IC_50_ 0.63 mg mL^−1^). Also, 166 and 167 showed 5-lipoxygenase inhibitory activity with IC_50_ values of 0.56 and 0.29 mg mL^−1^, respectively.^116^ 2α-Hydroxy-(28,29)-frido-olean-12 13, 21(22)-dien-20-propyl-21-hex-40(*E*)-enoate 168, and 2α-hydroxy-(28,29)-frido-olean-12 13, 21(22)-dien-20-prop-2-(*E*)-en-21-butanoate 169, frido-oleanene triterpenoids derivatives, 2α-hydroxy-8(17),12*E*,14-labdatriene 170, and 3β,6β,13α-trihydroxy-8(17),12*E*,14-labdatriene 171, labdane diterpenoids, were detected in an extract of *S. wightii*, were shown to be potential anti-hyperglycaemic pharmacophore leads to reduce the risk of elevated postprandial glucose levels. The oxygenated labdane diterpenoids displayed significantly lesser antioxidant and tyrosine phosphatase-1B inhibitory properties than those exhibited by the frido-oleanenes.^117^ 3′′-Isopropyl-3*c*-{3*b*-[(2-oxo-3,4-dihydro-2*H*-chromen-3-yl)methyl]butyl}-2′′-butenyl-3′-hydroxy-2′-(2′*b*-methoxy-2′-oxoethyl)-3′,4′-dihydro-2*H*-pyran-4′-carboxylate 172, 2*c*-methylbutyl-6-[6*c*-(benzoyloxy)propyl]-6-methyl-tetrahydro-2*H*-pyran-2-carboxylate 173, 6-{6*b*-[3′-(5′*a*-methyl propyl)-3′,4′-dihydro-2*H*-pyran-6′-yl]ethyl}-tetrahydro-2*H*-pyran-2-one 174 and 7-(7*c*-methylpentyl)-3,4,6,7,8,8*a*-hexahydro-2*H*-chromen-2-one 175 are hitherto undescribed O-heterocyclic derivatives with use natural antioxidant and antihypertensive functional food supplements and are also utilized as therapeutic leads in antihypertensive management as angiotensin-converting enzyme inhibitors (Fig. 10).^118^

## 55. Sargassum yezoense

Meroterphenol A 176, meroterphenol B 177, meroterphenol C 178, and meroterphenol D 179, plastoquinones, were detected in *S. yezoense*, with the structures of these compounds characterized by a 6-methyl-1,4-benzohydroquinone moiety with an oxygenated diterpenoic acid chain. Compounds 176–179 showed potent activation effects on the peroxisome proliferator-activated receptor gamma (PPARγ) (Fig. 10).^119^

## 56. Conclusion

**Fig. 11:**
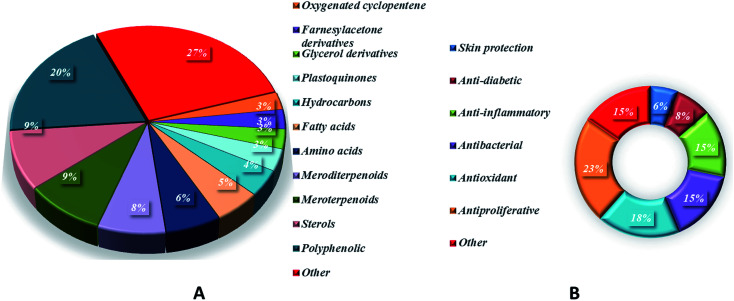
Secondary metabolites (A) and their reported bioactivity (B) produced by *Sargassum* species.

*Sargassum* is an important seaweed that is widely and excessively distributed in tropical and subtropical regions and has been reported to contain 537 species, with 358 of them accepted taxonomically. *Sargassum* species are used in many folk applications in human nutrition and are considered a rich source of vitamins, carotenoids, proteins, and minerals. Significant progress has been detected in the publication rates of studies mentioning the genus *Sargassum* over the past five years (Fig. 11), with many bioactive compounds reported with diverse chemical structures, from oxygenated cyclopentene, farnesylacetone, glycerol derivatives, plastoquinones, hydrocarbons, fatty acids, amino acids, to meroditerpenoids, meroterpenoids, sterols, polyphenolic, and sulfated polysaccharides (Fig. 2–10). According to the reported data concerning the phytochemical metabolites identified in the different *Sargassum* spp., the sulfated polysaccharides and polyphenolic compounds represent 27% and 20% of the major isolates, followed by meroterpenoids, sterols 9%, meroditerpenoids 8%, and amino acids 6%, reflecting their history as a nutrient in folk use (Fig. 11). These isolated compounds and/or extracts exhibit diverse biological activities; such as skin protection for the polysaccharide rich extract of *S. vachellianum*, and *S. fusiforme*, whereas the polysaccharide hardly absorbs UVA, UVB rays, and has shown considerable inhibition on tyrosinase (51.21%), as well as better moisture absorption (52.1%) and retention (63.24%) abilities. Also, *Sargassum* spp. contains mainly sulfated polysaccharides, and polyphenolic compounds (Fig. 11), reflecting major reports of their analgesic, anti-inflammatory 15%, antioxidant 18%, neuroprotective, anti-microbial 15%, anti-tumor 23% activities. The polysaccharide extracted from *S. asperifolium*, *S*. *hemiphyllum*, and *S. latifolium*, with *S. fulvellum*, *S. macrocarpum*, *S. horneri*, *S. patens*, *S. sagamianum*, and *S. serratifolium* possess anti-inflammatory activity, through a significant inhibition of nitric oxide generation, and LPS-induced TNF-α, IL-12 p40, IL-6, causing a strong inhibitory effect against NF-κB activation. While isonahocol E3 128, detected in *S*. *siliquastrum*, has functional antagonistic activities against ET-1 induced inflammation. Moreover, *S*. *angustifolium*, *S*. *boveanum*, and fucoidan *S. polycystum* extracts have been reported to have a cytotoxic effect against HeLa (cervical cancer) and MCF-7 (breast cancer) cells, where the cell survival is inversely proportional to the increase in the concentration of the extracts. Polysaccharides extracted from *S. latifolium* showed a selective cytotoxicity against lymphoblastic leukemia. 28*S*-Epoxy-24-ethylcholesterol 26 showed cytotoxic activity against MCF-7, HCT-8, 1A9, HOS, and PC-3 with IC_50_ values of 4.0, 8.8, 10.0, 10.0, and 7.2 μg mL^−1^, respectively. Sargachromanols A 109, B 110, and K 119 had strong cytotoxicity against AGS, HT-29, HT-1080, and MCF-7 cell lines, with IC_50_ values of 0.7, 6.1, 0.7, and 28.1 μg mL; 0.5, 1.0, 3.3, and 23.6 μg mL^−1^, and 5.7, 0.8, 1.8, and 10.3 μg mL^−1^, respectively. 28ξ-Dihydroxy-24-ethylcholesta-5,23*Z*-dien 21, and 24ξ-hydroperoxy-24-vinylcholesterol 24 were reported to have cytotoxic activities against HL-60, with IC_50_ values of 7.8 and 8.5 μg mL^−1^, respectively. While, fucosterol 5 and 24-ethylcholesta-4,24(28)-dien-3,6-dione 23 showed potent cytotoxic activities against P-388, with IC_50_ values of 0.7 and 0.8 μg mL^−1^, respectively. Sargachromanol R 126 had potent cytotoxic activity against AGS, HT-29, and HT-1080 cell lines, with IC_50_ values of 6.5, 3.4, and 13.9 μg mL^−1^, respectively, compared with paclitaxel and doxorubicin. Also, fucoidan extracted from *S. glaucescens*, *S. siliquosum*, *S. swartzii*, *S. tenerrimum*, and *S. pallidum* exhibited antioxidant activities in a dose-dependent manner against DPPH. A hot-water extract of *S. hemiphyllum* collected from the coast of Penghu County (Taiwan) had antioxidant activity and an immune-stimulating one. The antioxidant activity was showed to be increased related to a concentration <3.5 mg mL^−1^. Heteropolysaccharides extracted from *S. integerrimum* were shown to have neuroprotective and antioxidant activities. Sargachromanol A–P 109–124, isolated from *S*. *siliquastrum*, exhibited significant antioxidant activity in the DPPH assay. Sargachrominol 55, sargaquinoic acid 57, sargahydroquinoic acid 94, and sargathunbergol A 143 were detected in *S. thunbergii*, and showed antioxidant activities (EC_50_ values of 32, 27, 20, and 38 μg mL^−1^ respectively), compared to BHT (EC_50_ = 42 μg mL^−1^) and α-tocopherol (EC_50_ = 23 μg mL^−1^). Also, thunbergol A 144, and thunbergol B 145, isolated from *S. thunbergii*, were showed to scavenge DPPH radicals, with EC_50_ values of 30 and 31 μg mL^−1^, respectively. Additionally, an extract of *S. fulvellum* (SFEE) and grasshopper ketone 32 was shown to have an inhibitory effect on atopic dermatitis (AD)-like skin lesions in BALB/c mice by regulating immune mediators and cells and may be a potentially effective alternative therapy for AD. The sulfated polysaccharides from a hot-water crude extract of *S. kjellmanianum* showed an immune-regulatory effect, through the enhanced immune function of immunocytes and increased the proliferation rate of spleen lymphocytes and peritoneal macrophages up to 53.56% and 51.41%. *Sargassum* spp., isolated compounds and/or extracts, also exhibited anti-diabetic, fibrinolytic, anti-coagulant, hepatoprotective, and anti-viral activities. Despite the wide diversity in the reported biological activates for *Sargassum* spp., only 54/537 species, representing about 10.05% from the species accepted taxonomically, were detected as having chemically and pharmacologically relevant activities. Consequently, further studies on the remaining species, their constituents, and biological activities are needed to exploit their maximum therapeutic potential in the field of medicinal and pharmaceutical sciences for novel and fruitful applications.

In 2007, the genus *Sargassum* extracts were the focus of a patent as a cosmetic product (KR1020090064079A) for their activities in lowering photo-induced cytotoxicity, whitening effect, inhibiting MMP-1 biosynthesis in human fibroblasts, and promoting the synthesis of biosynthesis type 1 procollagen, thereby improving skin elasticity and fine wrinkles, and effectively inhibiting the photo-aging phenomenon promoted by ultraviolet rays, *etc.*^120^ Specially *Sargassum horneri* (KR1020120065631A), which has a deep relation to the photo-aging inhibitory effect on skin cells using the chromenes compound derived from it, increased the expression of collagen synthesis markers, such as procollagen and type I collagen in UV-A treated fibroblasts, and also enhanced elastin by modulating elastase activity, and thus could be used as a material and cosmetic for improving skin wrinkles and preventing wrinkles by UV.^121^ Also, *Sargassum natans* was part of a patent for removing gold ions from aqueous solution or suspension using a biomass-derived from it, and this process can be utilized to remove gold from industrial or natural waters.^122^

## Conflicts of interest

The authors declare there were no conflicts of interest.

## Supplementary Material

## References

[cit1] Liu L., Heinrich M., Myers S., Dworjanyn S. A. (2012). Towards a better understanding of medicinal uses of the brown seaweed *Sargassum* in traditional Chinese medicine: a phytochemical and pharmacological review. J. Ethnopharmacol..

[cit2] Mattio L., Payri C. E. (2011). 190 Years of *Sargassum* Taxonomy, Facing the Advent of DNA Phylogenies. Bot. Rev..

[cit3] Yende S. R., Harle U. N., Chaugule B. B. (2014). Therapeutic potential and health benefits of *Sargassum* species. Pharmacogn. Rev..

[cit4] Vaseghi G., Sharifi M., Dana N., Ghasemi A., Yegdaneh A. (2018). Cytotoxicity of *Sargassum angustifolium* Partitions against Breast and Cervical Cancer Cell Lines. Adv. Biomed. Res..

[cit5] Borazjani N. J., Tabarsa M., You S., Rezaei M. (2018). Purification, molecular properties, structural characterization, and immunomodulatory activities of water soluble polysaccharides from *Sargassum angustifolium*. Int. J. Biol. Macromol..

[cit6] Nasab S. B., Homaei A., Karami L. (2020). Kinetic of α-amylase inhibition by *Gracilaria corticata* and *Sargassum angustifolium* extracts and zinc oxide nanoparticles. Biocatal. Agric. Biotechnol..

[cit7] Moni S. S., Alam M. F., Makeen H. A., Alhazmi H. A., Sultan M., Siddiqui R., Jabeen A., Sanobar S., Alam M. S., Rehman Z. U., Elmobark M. E. (2019). Solvent extraction, spectral analysis and antibacterial activity of the bioactive crystals of *Sargassum aquifolium* (Turner) C. Agardh from Red Sea. Nat. Prod. Res..

[cit8] Bilan M. I., Ustyuzhanina N. E., Shashkov A. S., Thanh T. T. T., Bui M. L., Van Tran T. T., Usov A. I. (2017). Sulfated polysaccharides of the Vietnamese brown alga *Sargassum aquifolium* (Fucales, Sargassaceae). Carbohydr. Res..

[cit9] Camero M., Marinaro M., Lovero A., Elia G., Losurdo M., Buonavoglia C., Tempesta M. (2014). Polysaccharide extracts of the brown alga *Sargassum asperifolium* possess in vitro cancer chemopreventive properties. Nat. Prod. Res..

[cit10] Matloub A. A., Awad N. E. (2012). Phycochemistry of some *Sargassum* spp. and their cytotoxic and antimicrobial activities. Egypt. Pharm. J..

[cit11] Ayyad S. E. N., Sowellim S. Z., El-Hosini M. S., Abo-Atia A. (2003). The structural determination of a new steroidal metabolite from the brown alga *Sargassum asperifolium*. Z. Naturforsch..

[cit12] Tsuchiya N., Sato A., Haruyama H., Watanabe T., Iijima Y. (1998). Nahocols and isonahocols, endothelin antagonists from the brown alga, *Sargassum autumnale*. Phytochemistry.

[cit13] Noviendri D., Jaswir I., Salleh H. M., Taher M., Miyashita K., Ramli N. (2011). Fucoxanthin extraction and fatty acid analysis of *Sargassum binderi* and *S. duplicatum*. J. Med. Plants Res..

[cit14] Akbari V., Zafari S., Yegdaneh A. (2018). Anti-tuberculosis and cytotoxic evaluation of the seaweed *Sargassum boveanum*. Res. Pharm. Sci..

[cit15] Tang H. F., Yi Y. H., Yao X. S., Xu Q. Z., Zhang S. Y., Lin H. W. (2002). Bioactive steroids from the brown alga *Sargassum carpophyllum*. J. Asian Nat. Prod. Res..

[cit16] Tang H. F., Yi Y. H., Yao X. S., Xu Q., Zhang S., Lin H. (2003). A novel steroid for *Sargassum carpophyllum*. Zhongguo Haiyang Yaowu.

[cit17] Somasundaram S. N., Shanmugam S., Subramanian B., Jaganathan R. (2016). Cytotoxic effect of fucoidan extracted from *Sargassum cinereum* on colon cancer cell line HCT-15. Int. J. Biol. Macromol..

[cit18] Narayani S. S., Saravanan S., Ravindran J., Ramasamy M. S., Chitra J. (2019). In vitro anticancer activity of fucoidan extracted from *Sargassum cinereum* against Caco-2 cells. Int. J. Biol. Macromol..

[cit19] Baleta F. N., Bolaños J. M., Ruma O. C., Baleta A. N., Cairel J. D. (2017). Phytochemicals screening and antimicrobial properties of *Sargassum oligocystum* and *Sargassum crassifolium* extracts. J. Med. Plant.

[cit20] Sugiono S., Masruri M., Estiasih T., Widjarnako S. B. (2019). Structural and Rheological Characteristics of Alginate from *Sargassum cristaefolium* Extracted by Twin Screw Extruder. J. Aquat. Food Prod. Technol..

[cit21] Gamal-Eldeen A. M., Abo-Zeid M. A., Ahmed E. F. (2013). Anti-genotoxic effect of the *Sargassum dentifolium* extracts: prevention of chromosomal aberrations, micronuclei, and DNA fragmentation. Exp. Toxicol. Pathol..

[cit22] Madkour F. F., Khalil W. F., Dessouki A. A. (2012). Protective effect of ethanol extract of *Sargassum dentifolium* (Phaeophyceae) in carbon tetrachloride-induced hepatitis in rats. Int. J. Pharm. Pharm. Sci..

[cit23] Reddy P., Urban S. (2009). Meroditerpenoids from the southern Australian marine brown alga *Sargassum fallax*. Phytochem.

[cit24] Silva Costa L., Silva Telles C. B., Medeiros Oliveira R., Duarte Barreto Nobre L. T., Dantas-Santos N., Barros Gomes Camara R., Santana Santos Pereira Costa M., Almeida-Lima J., Melo-Silveira R. F., Lopes Albuquerque I. R., Leite E. L. (2011). Heterofucan from *Sargassum filipendula* Induces Apoptosis in HeLa Cells. Mar. Drugs.

[cit25] Chale-Dzul J., de Vaca R. P. C., Quintal-Novelo C., Olivera-Castillo L., Moo-Puc R. (2020). Hepatoprotective effect of a fucoidan extract from *Sargassum fluitans* Borgesen against CCl_4_-induced toxicity in rats. Int. J. Biol. Macromol..

[cit26] Kang B. K., Kim M. J., Ahn D. H. (2016). In vivo and in vitro inhibitory activity of an ethanolic extract of *Sargassum fulvellum* and its component grasshopper ketone on atopic dermatitis. Int. Immunopharmacol..

[cit27] Kang J. Y., Khan M. N. A., Park N. H., Cho J. Y., Lee M. C., Fujii H., Hong Y. K. (2008). Antipyretic, analgesic, and anti-inflammatory activities of the seaweed *Sargassum fulvellum* and *Sargassum thunbergii* in mice. J. Ethnopharmacol..

[cit28] Ina A., Hayashi K. I., Nozaki H., Kamei Y. (2007). Pheophytin a, a low molecular weight compound found in the marine brown alga *Sargassum fulvellum*, promotes the differentiation of PC12 cells. Int. J. Dev. Neurosci..

[cit29] Wu W., Hasumi K., Peng H., Hu X., Wang X., Bao B. (2009). Fibrinolytic Compounds Isolated from a Brown Alga, *Sargassum fulvellum*. Mar. Drugs.

[cit30] Kusumi T., Ishitsuka M., Iwashita T., Naoki H., Konno T., Kakisawa H. (1981). A novel type of glycerides bearing a methacrylic acid moiety from the brown alga, *Sargassum fulvellum*. Chem. Lett..

[cit31] Jia R. B., Li Z. R., Wu J., Ou Z. R., Zhu Q., Sun B., Lin L., Zhao M. (2019). Physicochemical properties of polysaccharide fractions from *Sargassum fusiforme* and their hypoglycemic and hypolipidemic activities in type 2 diabetic rats. Int. J. Biol. Macromol..

[cit32] Ye Y., Ji D., You L., Zhou L., Zhao Z., Brennan C. (2018). Structural properties and protective effect of *Sargassum fusiforme* polysaccharides against ultraviolet B radiation in hairless Kun Ming mice. J. Funct. Foods.

[cit33] Payghami N., Jamili S., Rustaiyan A., Saeidnia S., Nikan M., Gohari A. R. (2014). Alpha-amylase inhibitory activity and sterol composition of the marine algae, *Sargassum glaucescens*. Pharmacogn. Res..

[cit34] Huang C. Y., Wu S. J., Yang W. N., Kuan A. W., Chen C. Y. (2016). Antioxidant activities of crude extracts of fucoidan extracted from *Sargassum glaucescens* by a compressional-puffing-hydrothermal extraction process. Food Chem..

[cit35] Hwang P. A., Wu C. H., Gau S. Y., Chien S. Y., Hwang D. F. (2010). Antioxidant and immune-stimulating activities of hot-water extract from seaweed *Sargassum hemiphyllum*. J. Mar. Sci. Technol..

[cit36] Hwang P. A., Hung Y. L., Tsai Y. K., Chien S. Y., Kong Z. L. (2015). The brown seaweed *Sargassum hemiphyllum* exhibits α-amylase and α-glucosidase inhibitory activity and enhances insulin release *in vitro*. Cytotechnology.

[cit37] Hwang P. A., Chien S. Y., Chan Y. L., Lu M. K., Wu C. H., Kong Z. L., Wu C. J. (2011). Inhibition of lipopolysaccharide (LPS)-induced inflammatory responses by *Sargassum hemiphyllum* sulfated polysaccharide extract in RAW 264.7 macrophage cells. J. Agric. Food Chem..

[cit38] Wei M., Li S., Chen J., Lin Y. (2012). Chemical constituents of the brown alga *Sargassum henslowianum* collected from the South China Sea. Chem. Nat. Compd..

[cit39] Cuong H. D., Thuy T. T. T., Huong T. T., Ly B. M., Van T. T. T. (2015). Structure and hypolipidaemic activity of fucoidan extracted from brown seaweed *Sargassum henslowianum*. Nat. Prod. Res..

[cit40] Ale M. T., Maruyama H., Tamauchi H., Mikkelsen J. D., Meyer A. S. (2011). Fucose-Containing Sulfated Polysaccharides from Brown Seaweeds Inhibit Proliferation of Melanoma Cells and Induce Apoptosis by Activation of Caspase-3 *in vitro*. Mar. Drugs.

[cit41] Kim M. E., Jung Y. C., Jung I., Lee H. W., Youn H. Y., Lee J. S. (2015). Anti-Inflammatory Effects of Ethanolic Extract from *Sargassum horneri* (Turner) C. Agardh on Lipopolysaccharide-Stimulated Macrophage Activation via NF-κB Pathway Regulation. Immunol. Invest..

[cit42] Jayawardena T. U., Kim H. S., Sanjeewa K. A., Kim S. Y., Rho J. R., Jee Y., Ahn G., Jeon Y. J. (2019). *Sargassum horneri* and isolated 6-hydroxy-4,4,7a-trimethyl-5,6,7,7a-tetrahydrobenzofuran-2(4*H*)-one (HTT); LPS-induced inflammation attenuation via suppressing NF-κB, MAPK and oxidative stress through Nrf2/HO-1 pathways in RAW 264.7 macrophages. Algal Res..

[cit43] Nakayama M., Fukuoka Y., Nozaki H., Matsuo A., Hayashi S. (1980). Structure of (+)-kjellmanianone, a highly oxygenated cyclopentenone from the marine alga, *Sargassum kjellmanianum*. Chem. Lett..

[cit44] Nozaki H., Fukuoka Y., Matsuo A., Soga O., Nakayama M. (1980). Structure of sargassumlactam, a new *β, γ*-unsaturated-*γ*-lactam, from the marine alga *Sargassum kjellmanianum*. Chem. Lett..

[cit45] Yamamoto I., Takahashi M., Suzuki T., Seino H., Mori H. (1984). Antitumor effect of seaweeds. IV. Enhancement of antitumor activity by sulfation of a crude fucoidan fraction from *Sargassum kjellmanianum*. Jpn. J. Exp. Med..

[cit46] Ma Ww L. I. L., Zhou G. (2013). *In vitro* immunoregulatory and antitumor activity of sulfated polysaccharides from *Sargassum kjellmanianum*. Food Sci..

[cit47] Yende S. R., Harle U. N., Arora S. K., Pande V. B. (2018). Phytochemical screening and anticonvulsant activity of *Sargassum ilicifolium* (brown algae) in mice. J. Phytopharm..

[cit48] Jin W., Zhang W., Wang J., Yao J., Xie E., Liu D., Duan D., Zhang Q. (2014). A study of neuroprotective and antioxidant activities of heteropolysaccharides from six *Sargassum* species. Int. J. Biol. Macromol..

[cit49] Francesconi K. A., Edmonds J. S., Stick R. V., Skelton B. W., White A. H. (1991). Arsenic-containing ribosides from
the brown alga *Sargassum lacerifolium*: X-ray molecular structure of 2-amino-3-[5′-deoxy-5′-(dimethylarsinoyl) ribosyloxy]propane-1-sulphonic acid. J. Chem. Soc., Perkin Trans. 1.

[cit50] Gamal-Eldeen A. M., Ahmed E. F., Abo-Zeid M. A. (2009). *In vitro* cancer chemopreventive properties of polysaccharide extract from the brown alga, *Sargassum latifolium*. Food Chem. Toxicol..

[cit51] Abdel-Fattah A. F., Hussein M. M. D., Salem H. M. (1974). Some structural features of sargassan, a sulphated heteropolysaccharide from *Sargassum linifolium*. Carbohydr. Res..

[cit52] Kamei Y., Sueyoshi M., Hayashi K. I., Terada R., Nozaki H. (2009). The novel anti-propionibacterium acnes compound, sargafuran, found in the marine brown alga *Sargassum macrocarpum*. J. Antibiot..

[cit53] Tsang C. K., Ina A., Goto T., Kamei Y. (2005). Sargachromenol, a novel nerve growth factor-potentiating substance isolated from *Sargassum macrocarpum*, promotes neurite outgrowth and survival via distinct signaling pathways in PC12D cells. Neuroscience.

[cit54] Choi Y. K., Kim J., Lee K. M., Choi Y. J., Ye B. R., Kim M. S., Ko S. G., Lee S. H., Kang D. H., Heo S. J. (2017). Tuberatolide B suppresses cancer progression by promoting ROS-mediated inhibition of STAT3 signaling. Mar. Drugs.

[cit55] Manzoor Z., Mathema V. B., Chae D., Yoo E. S., Kang H. K., Hyun J. W., Lee N. H., Ko M. H., Koh Y. S. (2014). Extracts of the seaweed *Sargassum macrocarpum* inhibit the CpG-induced inflammatory response by attenuating the NF-κB pathway. Food Sci. Biotechnol..

[cit56] Zubia M., Payri C., Deslandes E. (2008). Alginate, mannitol, phenolic compounds and biological activities of two range-extending brown algae, *Sargassum mangarevense* and *Turbinaria ornata* (Phaeophyta: Fucales), from Tahiti (French Polynesia). J. Appl. Phycol..

[cit57] Bhaskar N. (2004). Growth inhibition of human pro-myelocytic leukemia (HL-60) cells by lipid extracts of marine alga *Sargassum marginatum* (Fucales, Phaeophyta) harvested off Goa (west coast of India) with special reference to fatty acid composition. Indian J. Mar. Sci..

[cit58] Ham Y. M., Kim K. N., Lee W. J., Lee N. H., Hyun C. G. (2010). Chemical constituents from *Sargassum micracanthum* and antioxidant activity. Int. J. Pharmacol..

[cit59] Mori J., Iwashima M., Wakasugi H., Saito H., Matsunaga T., Ogasawara M., Takahashi S., Suzuki H., Hayashi T. (2005). New plastoquinones isolated from the brown alga, *Sargassum micracanthum*. Chem. Pharm. Bull..

[cit60] Iwashima M., Mori J., Ting X., Matsunaga T., Hayashi K., Shinoda D., Saito H., Sankawa U., Hayashi T. (2005). Antioxidant and Antiviral Activities of Plastoquinones from the Brown Alga *Sargassum micracanthum*, and a New Chromene Derivative Converted from the Plastoquinones. Biol. Pharm. Bull..

[cit61] Kim C., Lee I. K., Cho G. Y., Oh K. H., Lim Y. W., Yun B. S. (2012). Sargassumol, a novel antioxidant from the brown alga *Sargassum micracanthum*. J. Antibiot..

[cit62] Shizuri Y., Matsukawa S., Ojika M., Yamada K. (1982). Two new farnesylacetone derivatives from the brown alga *Sargassum micracanthum*. Phytochem.

[cit63] Kusumi T., Ishitsuka M., Nomura Y., Konno T., Kakisawa H. (1979). New farnesylacetone derivatives from *Sargassum micracanthum*. Chem. Lett..

[cit64] Iwashima M. M., Tako N., Hayakawa T., Matsunaga T., Mori J., Saito H. (2008). New chromane derivatives isolated from the brown alga, *Sargassum micracanthum*. Chem. Pharm. Bull..

[cit65] Plouguerné E., Ioannou E., Georgantea P., Vagias C., Roussis V., Hellio C., Kraffe E., Stiger-Pouvreau V. (2010). Anti-microfouling activity of lipidic metabolites from the invasive brown alga *Sargassum muticum* (Yendo) Fensholt. Mar. Biotechnol..

[cit66] Lee J. A., Cho Y. R., Hong S. S., Ahn E. K. (2017). Anti-obesity activity of saringosterol isolated from *Sargassum muticum* (Yendo) Fensholt extract in 3T3-L1 cells. Phytother. Res..

[cit67] Devi D. V., Viswanathan P. (2019). Sulphated polysaccharide from *Sargassum myriocystum* confers protection against gentamicin-induced nephrotoxicity in adult zebrafish. Environ. Toxicol. Pharmacol..

[cit68] Peng Y., Xie E., Zheng K., Fredimoses M., Yang X., Zhou X., Wang Y., Yang B., Lin X., Liu J., Liu Y. (2013). Nutritional and Chemical Composition and Antiviral Activity of Cultivated Seaweed *Sargassum naozhouense* Tseng et Lu. Mar. Drugs.

[cit69] Peng Y., Huang R. M., Lin X. P., Liu Y. H. (2018). Norisoprenoids from the brown alga *Sargassum naozhouense* Tseng et Lu. Molecules.

[cit70] Mohammed A., Bissoon R., Bajnath E., Mohammed K., Lee T., Bissram M., John N., Jalsa N. K., Lee K. Y., Ward K. (2018). Multistage extraction and purification of waste *Sargassum natans* to produce sodium alginate: an optimization approach. Carbohydr. Polym..

[cit71] Permeh P., Saeidnia S., Mashinchian-Moradi A., Gohari A. R. (2012). Sterols from *Sargassum oligocystum*, a brown algae from the Persian Gulf, and their bioactivity. Nat. Prod. Res..

[cit72] Zandi K., Ahmadzadeh S., Tajbakhsh S., Rastian Z., Yousefi F., Farshadpour F., Sartavi K. (2010). Anticancer activity of *Sargassum oligocystum* water extract against human cancer cell lines. Eur. Rev. Med. Pharmacol. Sci..

[cit73] Ye H., Zhou C., Sun Y., Zhang X., Liu J., Hu Q., Zeng X. (2009). Antioxidant activities *in vitro* of ethanol extract from brown seaweed *Sargassum pallidum*. Eur. Food Res. Technol..

[cit74] Kim M. J., Kim M. J., Kim K. B. W. R., Park S. H., Choi H. D., Park S. Y., Kim J. H., Jang M. R., Im M. H., Ahn D. H. (2017). Anti-Inflammatory effect of *Sargassum patens* C. Agardh ethanol extract in LPS-induced RAW264. 7 cells and mouse ear edema. Microbiol. Biotechnol. Lett..

[cit75] Zhu W., Chiu L. C. M., Ooi V. E. C., Chan P. K. S., Ang Jr P. O. (2004). Antiviral property and mode of action of a sulphated polysaccharide from *Sargassum patens* against herpes simplex virus type 2. Int. J. Antimicrob. Agents.

[cit76] Brkljača R., Urban S. (2015). Chemical Profiling (HPLC-NMR andamp; HPLC-MS), Isolation, and Identification of Bioactive Meroditerpenoids from the Southern Australian Marine Brown Alga *Sargassum paradoxum*. Mar. Drugs.

[cit77] Qi S. H., Zhang S., Huang J. S., Xiao Z. H., Wu J., Long L. J. (2004). Glycerol derivatives and sterols from *Sargassum parvivesiculosum*. Chem. Pharm. Bull..

[cit78] Palanisamy S., Vinosha M., Marudhupandi T., Rajasekar P., Prabhu N. M. (2017). Isolation of fucoidan from *Sargassum polycystum* brown algae: structural characterization*, in vitro* antioxidant and anticancer activity. Int. J. Biol. Macromol..

[cit79] Lee C. W., Han J. S. (2012). Hypoglycemic effect of *Sargassum ringgoldianum* extract in STZ-induced diabetic mice. Prev. Nutr. Food Sci..

[cit80] Kim M. J., Jeong D. H., Ahn D. H. (2013). Anti-inflammatory activity of ethanolic extract of *Sargassum sagamianum* in RAW 264.7 cells. Food Sci. Biotechnol..

[cit81] Lee J. S., Lee H. A. (2019). *Sargassum sagamianum* extract protects INS-1 pancreatic β cells against high glucose-induced apoptosis. Cytotechnology.

[cit82] Chang H. W., Jang K. H., Lee D., Kang H. R., Kim T. Y., Lee B. H., Choi B. W., Kim S., Shin J. (2008). Monoglycerides from the brown alga *Sargassum sagamianum*: isolation, synthesis, and biological activity. Bioorg. Med. Chem. Lett..

[cit83] Choi B. W., Ryu G., Park S. H., Kim E. S., Shin J., Roh S. S., Shin H. C., Lee B. H. (2007). Anticholinesterase activity of plastoquinones from *Sargassum sagamianum*: lead compounds for Alzheimer's disease therapy. Phytother. Res..

[cit84] Hur S., Lee H., Kim Y., Lee B. H., Shin J., Kim T. Y. (2008). Sargaquinoic acid and sargachromenol, extracts of *Sargassum sagamianum*, induce apoptosis in HaCaT cells and mice skin: Its potentiation of UVB-induced apoptosis. Eur. J. Pharmacol..

[cit85] Horie S., Tsutsumi S., Takada Y., Kimura J. (2008). Antibacterial Quinone Metabolites from the Brown Alga, *Sargassum sagamianum*. Bull. Chem. Soc. Jpn..

[cit86] Joung E. J., Kwon M., Gwon W. G., Cao L., Lee S. G., Utsuki T., Wakamatsu N., Kim J. I., Kim H. R. (2020). Meroterpenoid-rich fraction of the ethanol extract of *Sargassum serratifolium* suppresses collagen-induced rheumatoid arthritis in DBA/1J mice via inhibition of nuclear factor κB activation. Mol. Nutr. Food Res..

[cit87] Kim Y. H., Kim J. H., Kim D. H., Kim S. H., Kim H. R., Kim Y. M. (2016). Synergistic antimicrobial effect of *Sargassum serratifolium* (C. Agardh) C. Agardh extract against human skin pathogens. Korean J. Food Sci. Technol..

[cit88] Kwon M., Lim S. J., Lee B., Shin T., Kim H. R. (2018). Ethanolic extract of *Sargassum serratifolium* inhibits adipogenesis in 3T3-L1 preadipocytes by cell cycle arrest. J. Appl. Phycol..

[cit89] Jang K. H., Lee B. H., Choi B. W., Lee H. S., Shin J. (2005). Chromenes from the brown alga *Sargassum siliquastrum*. J. Nat. Prod..

[cit90] Lee J. I., Park B. J., Kim H., Seo Y. (2014). Isolation of Two New Meroterpenoids from *Sargassum siliquastrum*. Bull. Korean Chem. Soc..

[cit91] Cho S. H., Cho J. Y., Kang S. E., Hong Y. K., Ahn D. H. (2008). Antioxidant activity of mojabanchromanol, a novel chromene, isolated from brown alga *Sargassum siliquastrum*. J. Environ. Biol..

[cit92] Sah S. K., Kim B. H., Park G. T., Kim S., Jang K. H., Jeon J. E., Shin J., Kim T. Y. (2013). Novel isonahocol E3 exhibits anti-inflammatory and anti-angiogenic effects in endothelin-1-stimulated human keratinocytes. Eur. J. Pharmacol..

[cit93] Lee J. I., Kwak M. K., Park H. Y., Seo Y. (2013). Cytotoxicity of meroterpenoids from *Sargassum siliquastrum* against human cancer cells. Nat. Prod. Commun..

[cit94] Jung M., Jang K. H., Kim B., Lee B. H., Choi B. W., Oh K. B., Shin J. (2008). Meroditerpenoids from the brown alga *Sargassum siliquastrum*. J. Nat. Prod..

[cit95] Nagappan H., Pee P. P., Kee S. H. Y., Ow J. T., Yan S. W., Chew L. Y., Kong K. W. (2017). Malaysian brown seaweeds *Sargassum siliquosum* and *Sargassum polycystum*: Low density lipoprotein (LDL) oxidation, angiotensin converting enzyme (ACE), α-amylase, and α-glucosidase inhibition activities. Food Res. Int..

[cit96] Glombitza K. W., Keusgen M. (1995). Fuhalols and deshydroxyfuhalols from the brown alga *Sargassum spinuligerum*. Phytochem.

[cit97] Glombitza K. W., Keusgen M., Hauperich S. (1997). Fucophlorethols from the brown algae *Sargassum spinuligerum* and *Cystophora torulosa*. Phytochem.

[cit98] Keusgen M., Glombitza K. W. (1997). Pseudofuhalols from the brown alga *Sargassum spinuligerum*. Phytochem.

[cit99] Vijayabaskar P., Vaseela N., Thirumaran G. (2012). Potential antibacterial and antioxidant properties of a sulfated polysaccharide from the brown marine algae *Sargassum swartzii*. Chin. J. Nat. Med..

[cit100] Dinesh S., Menon T., Hanna L. E., Suresh V., Sathuvan M., Manikannan M. (2016). In vitro anti-HIV-1 activity of fucoidan from *Sargassum swartzii*. Int. J. Biol. Macromol..

[cit101] Vijayabaskar P., Vaseela N. (2012). In vitro antioxidant properties of sulfated polysaccharide from brown marine algae *Sargassum tenerrimum*. Asian Pac. J. Trop. Dis..

[cit102] Shibata Y., Morita M. (1988). A novel, trimethylated arseno-sugar isolated from the brown alga *Sargassum thunbergii*. Agric. Biol. Chem..

[cit103] Seo Y. W., Park K. E., Nam T. J. (2007). Isolation of a new chromene from the brown alga *Sargassum thunbergii*. Bull. Korean Chem. Soc..

[cit104] Seo Y., Park K. E., Kim Y. A., Lee H. J., Yoo J. S., Ahn J. W., Lee B. J. (2006). Isolation of tetraprenyltoluquinols from the brownalga *Sargassum thunbergii*. Chem. Pharm. Bull..

[cit105] Kim Y. H., Kim E. H., Lee C., Kim M. H., Rho J. R. (2007). Two new monogalactosyl diacylglycerols from brown alga *Sargassum thunbergii*. Lipids.

[cit106] He W. F., Yao L. G., Liu H. L., Guo Y. W. (2014). Thunberol, a new sterol from the Chinese brown alga *Sargassum thunbergii*. J. Asian Nat. Prod. Res..

[cit107] Kang M. C., Ding Y., Kim E. A., Choi Y. K., De Araujo T., Heo S. J., Lee S. H. (2017). Indole derivatives isolated from brown alga *Sargassum thunbergii* inhibit adipogenesis through AMPK activation in 3T3-L1 preadipocytes. Mar. Drugs.

[cit108] Park K. E., Kim Y. A., Jung H. A., Lee H. J., Ahn J. W., Lee B. J., Seo Y. W. (2004). Three norisoprenoids from the brown alga *Sargassum thunbergii*. J. Korean Chem. Soc..

[cit109] Cai Y. P., Xie C. B., Wang B. C., Li P. L., Li B. F. (2010). Two new resorcinols from *Sargassum thunbergii*. J. Asian Nat. Prod. Res..

[cit110] Kim K. B. W. R., Kim M. J., Ahn D. H. (2014). Lipase inhibitory activity of chlorophyll a, isofucosterol and saringosterol isolated from chloroform fraction of *Sargassum thunbergii*. Nat. Prod. Res..

[cit111] Lee J. B., Takeshita A., Hayashi K., Hayashi T. (2011). Structures and antiviral activities of polysaccharides from *Sargassum trichophyllum*. Carbohydr. Polym..

[cit112] Fenoradosoa T. A., Ali G., Delattre C., Laroche C., Petit E., Wadouachi A., Michaud P. (2010). Extraction and characterization of an alginate from the brown seaweed *Sargassum turbinarioides* Grunow. J. Appl. Phycol..

[cit113] Jesumani V., Du H., Pei P., Aslam M., Huang N. (2020). Comparative study on skin protection activity of polyphenol-rich extract and polysaccharide-rich extract from *Sargassum vachellianum*. PLoS One.

[cit114] Xu S. H., Cen Y. Z., Li Y. L., Xu S. Y. (1999). A novel eleven-membered heterocyclic compound from algae *Sargassum vachellianum* CA131:283698. Chin. Chem. Lett..

[cit115] Arunkumar K., Selvapalam N., Rengasamy R. (2005). The antibacterial compound sulphoglycerolipid 1-0 palmitoyl-3-0(6′-sulpho-alpha-quinovopyranosyl)-glycerol from *Sargassum wightii* Greville (Phaeophyceae). Bot. Mar..

[cit116] Maneesh A., Chakraborty K. (2017). Unprecedented antioxidative and anti-inflammatory aryl polyketides from the brown seaweed *Sargassum wightii*. Food Res. Int..

[cit117] Maneesh A., Chakraborty K. (2017). Previously undescribed frido oleanenes and oxygenated labdanes from the brown seaweed *Sargassum wightii* and their protein tyrosine phosphatase-1B inhibitory activity. Phytochem.

[cit118] Maneesh A., Chakraborty K. (2018). Previously undescribed antioxidative O-heterocyclic angiotensin converting enzyme inhibitors from the intertidal seaweed *Sargassum wightii* as potential antihypertensives. Food Res. Int..

[cit119] Kim M. C., Kwon H. C., Kim S. N., Kim H. S., Um B. H. (2011). Plastoquinones from *Sargassum yezoense*; Chemical Structures and Effects on the Activation of Peroxisome Proliferator-Activated Receptor Gamma. Chem. Pharm. Bull..

[cit120] https://patents.google.com/patent/KR20110006444A/en

[cit121] https://patents.google.com/patent/KR20130142424A/en

[cit122] https://patents.justia.com/patent/4769223#history

